# Global estimates of paediatric tuberculosis incidence in 2013–19: a mathematical modelling analysis

**DOI:** 10.1016/S2214-109X(21)00462-9

**Published:** 2021-12-08

**Authors:** Sita Yerramsetti, Ted Cohen, Rifat Atun, Nicolas A Menzies

**Affiliations:** Department of Global Health and Population (S Yerramsetti BA, Prof R Atun PhD, N A Menzies PhD) and Center for Health Decision Science (N A Menzies), Harvard T H Chan School of Public Health, Boston, MA, USA; Department of Epidemiology of Microbial Diseases, Yale School of Public Health, New Haven, CT, USA (Prof T Cohen DPH)

## Abstract

**Background:**

Many children who develop tuberculosis are thought to be missed by diagnostic and reporting systems. We aimed to estimate paediatric tuberculosis incidence and underreporting between 2013 and 2019 in countries representing more than 99% of the global tuberculosis burden.

**Methods:**

We developed a mathematical model of paediatric tuberculosis natural history, accounting for key mechanisms and risk factors for infectious exposure (HIV, malnutrition, and BCG non-vaccination), the probability of infection given exposure, and progression to disease among infected individuals. We extracted paediatric population estimates from UN Population Division data, and we used WHO estimates for adult tuberculosis incidence rates. We parameterised this model for 185 countries and calibrated it using data from countries with stronger case detection and reporting systems. Using this model, we estimated trends in paediatric incidence, and the proportion of these cases that are diagnosed and reported (case detection ratio [CDR]) for each country, age group, and year.

**Findings:**

For 2019, we estimated 997 500 (95% credible interval [CrI] 868 700–1 163 100) incident tuberculosis cases among children, with 481 000 cases (398 400–587 400) among those aged 0–4 years and 516 500 cases (442 900–608 000) among those aged 5–14 years. The paediatric CDR was estimated to be lower in children aged 0–4 years (41%, 95% CrI 34–50) than in those aged 5–14 years (63%, 53–75) and varied widely between countries. Estimated CDRs increased substantially over the study period, from 18% (15–20) in 2013 to 53% (45–60) in 2019, with improvements concentrated in the Eastern Mediterranean, South-East Asia, and Western Pacific regions. Over the study period, global incidence was estimated to have declined slowly at an average annual rate of 1·52% (1·42–1·66).

**Interpretation:**

Paediatric tuberculosis causes substantial morbidity and mortality, and these data indicate that cases (and, thus, probably associated mortality) are currently substantially underreported. These findings reinforce the need to ensure prompt diagnosis and care for children developing tuberculosis, strengthen reporting systems, and invest in research to develop more accurate and easy-to-use diagnostics for paediatric tuberculosis in high-burden settings.

**Funding:**

National Institutes of Health.

## Abstract (summary)

Editorial note: the *Lancet* Group takes a neutral position with respect to territorial claims in published maps and institutional affiliations.

## Introduction

Tuberculosis causes more than 1 million deaths each year.^1^ Although most tuberculosis deaths occur in older age groups, young children (aged 0–2 years) represent a special risk group as they have higher risks of developing tuberculosis if infected, with rapid disease progression and high case fatality.^2^ For this reason, strengthening paediatric tuberculosis prevention, detection, and treatment has been a special focus of efforts to improve tuberculosis control globally.^3^

Estimates from the past few years suggest that a third of all individuals with tuberculosis do not receive prompt diagnosis and treatment.^4,5^ Several factors make detection of paediatric tuberculosis particularly difficult. One is the low prevalence of tuberculosis compared with that of other childhood illnesses with similar presentation,^6^ resulting in low clinical suspicion of tuberculosis in children with persistent cough and fever. Another obstacle is the reliance on sputum smear microscopy for tuberculosis diagnosis in many high-burden settings. Obtaining sputum samples from young children is difficult, and pulmonary tuberculosis can have very low bacterial loads in this population. Consequently, smear microscopy has poor sensitivity in children—as low as 1% in children aged 0–4 years, and 14% in those aged 5–14 years^7^—and diagnosis relies heavily on clinical presentation. Although improved algorithms for clinical diagnosis have been developed,^8,9^ these new approaches still have limited sensitivity and specificity, such that children without tuberculosis could receive an incorrect positive diagnosis. Due to these challenges, many children who develop tuberculosis do not receive prompt and accurate diagnosis.

Undiagnosed tuberculosis is estimated to be fatal in 20% of paediatric cases—over 40% of cases in children aged 0–4 years^10^—and individuals surviving the disease episode can have long-term disability.^11^ In addition to the individual health consequences, under-ascertainment of paediatric tuberculosis at a population level can obscure the magnitude of the disease burden. This impedes resource allocation, intervention development, and programmatic efforts to address paediatric tuberculosis.^12^ Because few empirical alternatives exist for assessing paediatric tuberculosis burden, the quality of paediatric case detection within individual countries is difficult to judge, since low numbers of paediatric tuberculosis notifications could reflect low disease incidence, poor detection, or poor reporting. Although the quality of case reporting has improved since 2005,^12^ empirical approaches to quantify the completeness of case detection are still needed.

Given the challenges with case detection and surveillance, approaches have been developed to estimate paediatric tuberculosis incidence through mathematical modelling. In 2014, Jenkins and colleagues described a method that imputed missing smear-negative notifications by use of smear-positive paediatric notifications and age-stratified estimates of smear-positivity among confirmed tuberculosis cases.^13^ In the same year, Dodd and colleagues described two approaches for estimating paediatric tuberculosis incidence in 22 high-tuberculosis burden countries. The first approach (community model) specified a linear model relating adult disease prevalence to paediatric *Mycobacterium tuberculosis* infection, with model parameters derived from observational data. The second approach used mechanistic modelling to estimate infection rates through household exposures, using Demographic Household Survey (DHS) data.^14^ Both approaches used a natural history model to translate paediatric infections into incidence estimates. These modelled analyses, and an update to the Dodd community model in 2016,^15^ estimated close to 1 million annual paediatric tuberculosis cases—substantially greater than paediatric tuberculosis notifications. The Dodd community model is now used by WHO to inform estimated patterns of tuberculosis incidence across age groups, with 1·2 million global paediatric tuberculosis cases estimated for 2019.^1,16^ Separately, the Institute of Health Metrics and Evaluation (IHME) produces tuberculosis incidence estimates for the Global Burden of Diseases, Injuries, and Risk Factors Study (GBD) by use of a comprehensive meta-analytical framework to synthesis data on deaths, notifications, and other explanatory variables.^17^

These modelling studies have shown large gaps between total paediatric tuberculosis incidence and the number of cases diagnosed and reported each year. To further characterise this gap, we developed a novel mathematical model of paediatric tuberculosis incidence that extends earlier approaches. The model explicitly accounts for undernutrition, a risk factor to which an estimated 27% of tuberculosis cases in high-risk countries have been attributed,^18^ and differences in paediatric tuberculosis exposure based on respiratory mixing matrices published in 2020.^19^ This model is also calibrated to paediatric notification data in countries with stronger case detection and reporting systems, providing an additional source of identification for incidence estimates. With this approach, we aimed to estimate paediatric tuberculosis incidence during 2013–19 in the 185 countries with more than 99% of the global tuberculosis burden. We compared these incidence estimates with reported notifications to estimate the completeness of case detection across countries and time, and report how the results from this new method compare with earlier approaches.

## Methods

### Study model

We developed a mathematical model describing the events that lead to childhood tuberculosis disease, accounting for differences in adult tuberculosis prevalence and other factors that determine *M tuberculosis* exposure risks, the probability of infection after exposure, and the probability of tuberculosis disease among infected individuals (appendix 1 p 5). The model stratifies the paediatric population by country, year, and age group (0–4 years and 5–14 years).

### Data inputs

We extracted population estimates (*P*_*jkt*_) by paediatric age group (*j*), country (*k*), and year (*t*) from UN Population Division data.^20^ For adult tuberculosis incidence rates (*r*_*ikt*_), we used WHO estimates of absolute incidence by adult age group (*i*), country, and year.^21^ Because age-stratified incidence estimates were only available from 2018 onwards, we estimated age-stratified values for 2013–17 by multiplying the 2018 age distribution by the absolute number of adult cases in each preceding year. We calculated incidence rates by dividing absolute incidence by population size for each age group, country, and year. For the proportion of adult incident cases detected by country and year (*S*_*kt*_), we used WHO country-level case detection ratios (CDRs),^21^ assuming that these values approximate the CDR for adult age groups. We extracted the prevalence of paediatric HIV and undernutrition (*u*_*jkt*_, operationalised as protein-energy malnutrition [moderate or severe acute wasting]) from GBD 2019^22^ and paediatric antiretroviral therapy coverage from UNAIDS.^23^ We used HIV prevalence and antiretroviral therapy coverage to calculate the prevalence of treated (*t*_*jkt*_) and untreated (*h*_*jkt*_) HIV. We extracted BCG vaccination coverage (*v*_*jkt*_) from WHO–UNICEF estimates.^24^ For the number of contacts between paediatric and adult age groups, we used recently published respiratory contact matrices for 177 countries.^19^ For countries omitted from that analysis, we estimated contact matrices by taking element-wise averages of contact matrices for countries in the same WHO region. We validated this approach by imputing data for all non-missing countries for which the mean absolute percentage error was 17%. Input data are given in appendix 1 (pp 21–30).

### Model parameters

We used Bayesian methods to implement the analysis.^25^ We specified probability distributions representing available evidence for each parameter. For the duration of untreated tuberculosis disease (*d*_*untx*_), we assumed a mean of 3·00 years (range 2·50–3·50), on the basis of historical cohort data.^26^ For the proportion of time spent in subclinical disease (*f*), we assumed a mean fraction of 0·25 (0·17–0·33), equivalent to 9 months (6–12), on the basis of research describing a large proportion of individuals who were asymptomatic among prevalent tuberculosis cases.^27^ For the probability of infection per infectious contact (*b*), we divided an estimate of the effective contact rate (12 [6–15], the annual number of infections caused by an infectious case in a susceptible population)^28,29^ by the average annual number of contacts for children. For the probability of progression to tuberculosis disease for newly-infected individuals (*a*_*j*_), we used a value of 0·19 (range 0·08–0·37) for children aged 0–4 years, and 0·09 (0·05–0·16) for children aged 5–14 years.^30^ For the rate of contact saturation (*q*), whereby overall transmission is reduced by infectious contacts being concentrated within a small number of individuals, we specified a weak previous distribution centred at 0·50 (range 0·10–0·90) and estimated this value through calibration. For the risk ratio of disease with untreated HIV (*m*^*h*^), we specified a value of 7·90 (range 4·50–13·70).^31^ For the risk ratio of disease with treated HIV compared with untreated HIV (*m*^*t*^), we specified a value of 0·30 (0·21–0·39).^31^ For the risk ratio of disease with underweight (*m*^*u*^), we specified a value of 4·0 (2·0–6·0), on the basis of a review of observational cohort studies.^32^ For the risk ratio of disease with BCG vaccination (*m*^*V*^_*k*_), we specified country-specific values based on evidence showing greater BCG vaccine effectiveness at higher latitudes.^33^ For each parameter, we specified probability distributions to produce mean values and 95% uncertainty interval widths matching the values given previously (appendix 1 p 6). Other inputs and analytical code are provided in a Dataverse online repository.

### Model calibration

Calibration data consisted of paediatric case notifications for a subset of countries identified as having better paediatric case detection. To create this subset, we selected countries with high CDR values (CDR of 0·85 or higher averaged over the study period, which comprised 75 countries) and age-standardised death-to-notification ratios in the lowest quintile of countries over the study period (37 countries), under the assumption that countries with low deaths relative to notifications would have more complete case detection. We derived age-standardised death-to-notification ratios using tuberculosis mortality estimates from GBD 2019.^22^ We used the countries classified as having more complete case detection according to both these approaches—27 countries in total—for model calibration (appendix p 3).

We specified negative binomial likelihood functions for the calibration data, with reported paediatric notifications compared with the model estimate for the same quantity (incidence multiplied by fraction detected) for each country, age group, and year. We allowed all model parameters to vary according to their defined probability distributions (appendix 1 p 6). We used Hamiltonian Monte Carlo^34^ to obtain samples from the posterior distribution (appendix 1 pp 7–8), with the model fit to all calibration data simultaneously. Using the fitted model, we estimated the distribution of results for all countries and outcomes of interest. We summarised results as the mean value for each of these distributions. Further details are provided in appendix 1 (pp 3–4), including a comparison of fitted values with calibration targets (p 9). Processing of data and results was undertaken in R, version 4.0.2, and model fitting was done with Stan.

### Outcomes

We estimated results for all countries with available data over the study period (185 countries; appendix 1 pp 10–14), representing more than 99% of global paediatric notifications. For each country, we estimated paediatric tuberculosis incidence for the age groups of 0–4 years and 5–14 years, from 2013 to 2019. We also divided reported notifications by incidence values to estimate the proportion of paediatric tuberculosis cases diagnosed and reported (the paediatric CDR) by country, year, and age group. For strata in which reported cases were greater than modelled incidence, we assumed the reported value to be correct, and we inflated the distribution of incidence results so that the mean incidence estimate matched total notifications. We report these countries as having a CDR point estimate of 100%. Additionally, we recomputed results with the prevalence of each risk factor (HIV, malnutrition, and BCG non-vaccination) set to 0, to report the contribution of each factor to total incidence estimates.

### Sensitivity analysis

We tested the robustness of the analysis to alternative assumptions.^35^ First, we estimated the sensitivity of results to individual parameter changes, by setting each parameter to different fixed values spanning the range of the original parameter distribution and recalibrating other parameters conditional on that value. This analysis describes the relationship between each parameter and the incidence estimate, while still fitting the calibration data. Second, we computed uncertainty intervals quantifying the implications of multivariate uncertainty in model parameters, reported as equal-tailed 95% credible intervals (CrIs). Third, we compared results with 2019 paediatric incidence estimates produced by WHO and IHME,^22^ to describe differences between these three sets of estimates. Finally, to understand the sensitivity of results to different contact patterns, we re-estimated results with contact matrices set to the global average.

### Role of the funding source

The funder had no role in study design, implementation, data collection, data analysis, data interpretation, or writing of the report.

## Results

**Figure 1:**
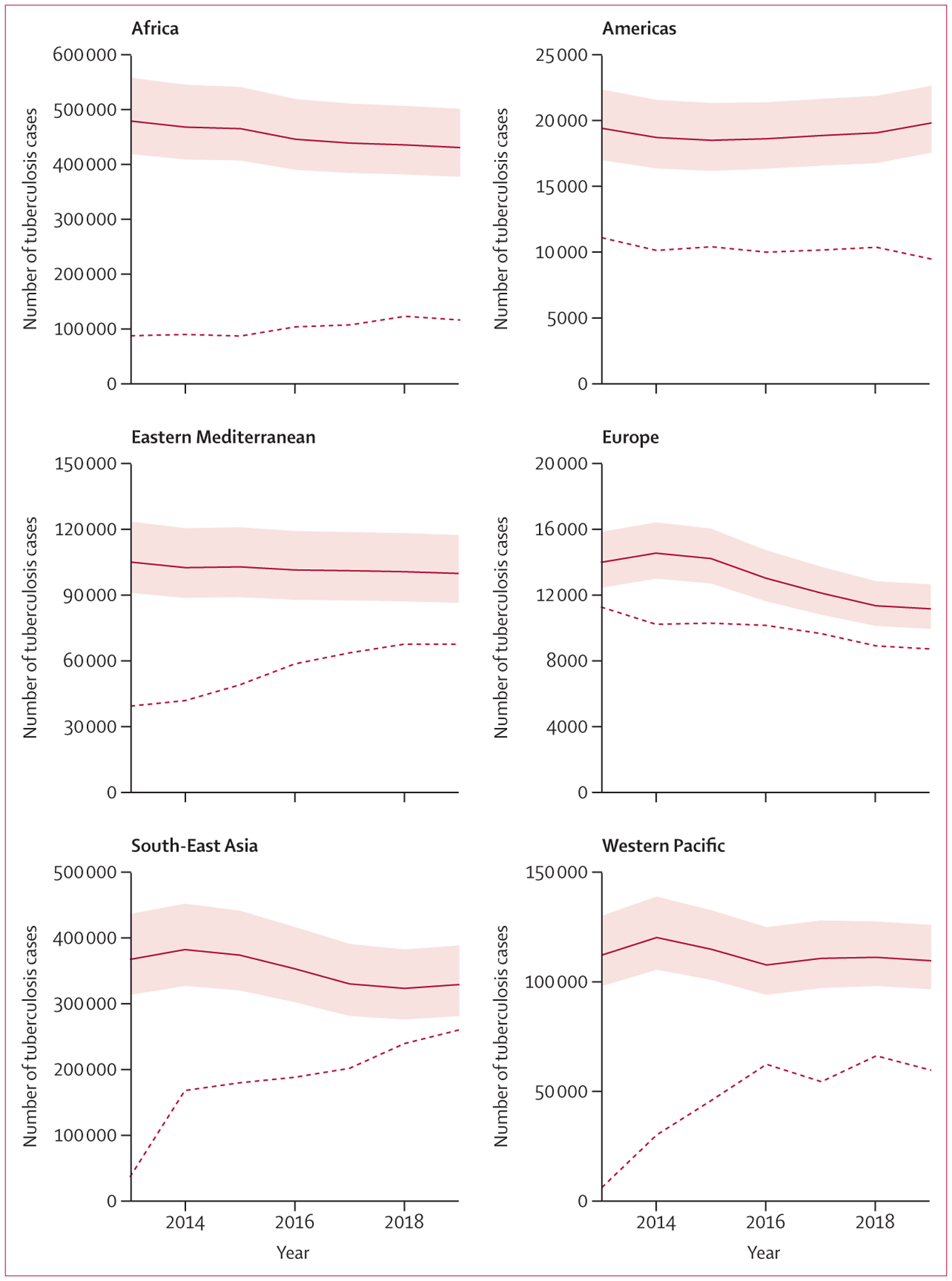
Trends in paediatric incidence estimates compared with total case notifications for each WHO region, 2013–19 Solid lines and shaded bands represent modelled point estimates and 95% credible interval for tuberculosis cases in each WHO region. Dashed lines represent reported case notifications.

We estimated global paediatric tuberculosis incidence to be 997 500 (95% CrI 868 700–1163 100) in 2019, summed across the 185 countries in the analysis. Table 1 summarises regional and worldwide estimates of paediatric tuberculosis incidence in 2019, and figure 1 compares regional time trends of incidence estimates and reported tuberculosis cases over 2013–19. Detailed incidence estimates are given in appendix 1 (pp 10–15) and appendix 2.

**Figure 2:**
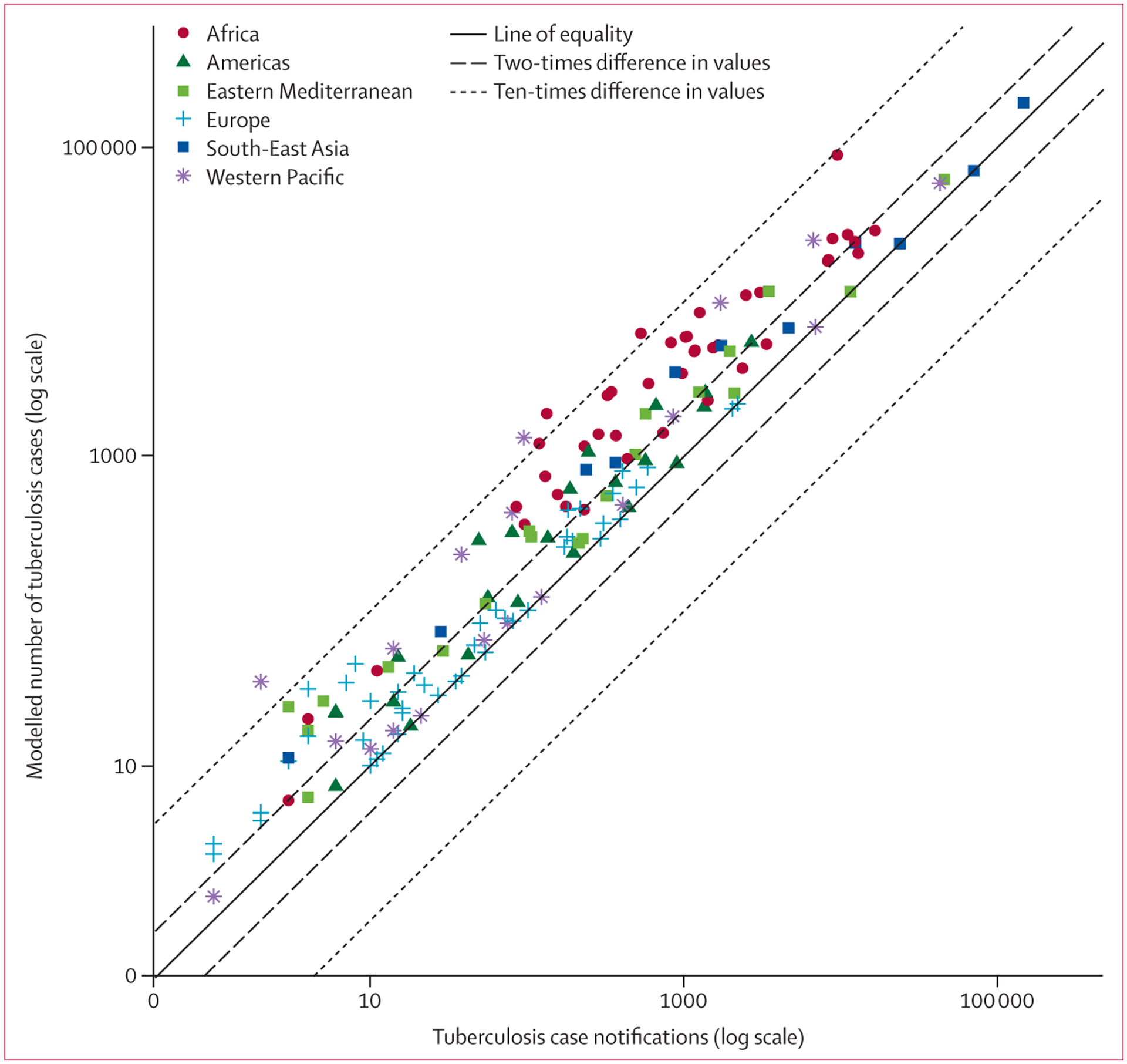
Country-level paediatric tuberculosis incidence estimates compared with case notifications in 2019 Countries with no paediatric case notifications in 2019 (25 countries) were excluded from the plot. Of the countries with estimated paediatric incidence higher than 100 cases, eight countries had case detection ratio values lower than 15% (Burkina Faso [7%], Laos [7%], Niger [8%], Guinea-Bissau [10%], Nigeria [11%], Burundi [13%], Mali [13%], and Cameroon [15%]), excluding countries with no reported paediatric case notifications.

In total, 520 818 paediatric cases were reported in 2019 for the 185 countries in the analysis, producing a global CDR of 53% (45–60) when compared with the modelled incidence estimates. Over the study period, global incidence was estimated to have declined slowly at an average annual rate of 1·52% (1·42–1·66). By age group, these CDR values were 41% (34–50) for children aged 0–4 years and 63% (53–75) for those aged 5–14 years. Globally, the CDR was estimated to increase substantially over the study period, from a low of 18% (15–20) in 2013. We compared modelled incidence estimates with reported case notifications in 2019 for all countries, stratified by WHO region (figure 2; appendix 1 pp 10–14). Countries in the African region had the lowest average CDRs (shown by distance above the line of equality), although there is wide variation within each region.

We summarised paediatric incidence and CDR estimates for the 30 countries with the highest burden of tuberculosis in 2019 according to WHO (table 2). For this group, several countries (Indonesia, Myanmar, Papua New Guinea, and Russia) were estimated to have high levels of diagnosis and reporting, whereas Cambodia, Democratic Republic of the Congo, Nigeria, and Vietnam were estimated to have diagnosed and reported fewer than 20% of paediatric tuberculosis cases in 2019. Detailed CDR estimates are presented in appendix 1 (pp 10–14) and appendix 2.

We compared our results with other incidence estimates. The WHO estimate for paediatric tuberculosis incidence in 2019 for the 185 countries included in our analysis was 1 175 000 cases.^21^ This is 18% (95% CrI 1–35) greater than our estimate, largely due to substantially higher WHO estimates for a small number of countries. In 2019, India—the highest-incidence country in both sets of estimates—was estimated to have 193 000 (95% CrI 164 300–228 900) cases in our analysis (table 2), and 333 000 cases in WHO analyses. The gap for India alone represents 78% of the difference in total paediatric incidence between our estimates and WHO estimates. For 129 (70%) of 185 countries, our incidence estimate was greater than the corresponding WHO estimate. For the same year, IHME estimated 851 000 paediatric cases in the included countries, 14% (95% CrI 2–27) lower than our estimate. Our estimates were higher than the corresponding IHME estimate^22^ for 113 (61%) of 185 countries, including India, for which IHME estimated 128 000 paediatric cases in 2019. In appendix 1, we provide country-specific comparisons between the three sets of incidence estimates for 2019 (p 16) and a comparison of estimates for the 30 countries categorised as having high tuberculosis burden by WHO (p 17).^1^

We estimated the contribution of each modelled risk factor to paediatric tuberculosis incidence. Globally, HIV was estimated to be responsible for 0·7% (95% CrI 0·3–1·3) of total incidence, malnutrition for 12·7% (5·8–20·5), and BCG non-vaccination for 13·5% (9·5–17·8); collectively, these three risk factors were estimated to be responsible for 25·1% (17·8–33·0) of total incidence (appendix p 18). Table 3 shows estimated paediatric tuberculosis cases attributable to each factor by world region.

We did sensitivity analyses for each model parameter (appendix 1 p 19), with each parameter varied across the ranges (appendix 1 p 6). These analyses showed the total incidence estimate to be relatively robust to individual parameter changes once the analysis is constrained to fit the calibration data. The parameter with the largest influence on global incidence was *q*, which moderates the effect of contact saturation. Lower values of *q* (indicating a smaller role for contact saturation) produced higher global incidence estimates. How incidence estimates changed when we re-estimated the analysis by removing inter-country differences in contact patterns can be seen in the appendix (p 20). Estimates for most countries were robust to this change (1·9% average percentage change).

## Discussion

In this study, we developed a novel mathematical model of paediatric tuberculosis exposure, infection, and progression. This model extends earlier approaches to account for undernutrition as a key risk factor for tuberculosis, incorporate new evidence on respiratory contact patterns, and use evidence from countries with stronger case detection and reporting to calibrate incidence estimates. Using this model, we estimated more than 990 000 incident tuberculosis cases among children in 2019, substantially higher than the roughly 520 000 cases diagnosed and reported to WHO for the same year. 2019 represented the year with the highest level of case detection and reporting in our analysis and, over the 7-year period of our study, the Eastern Mediterranean, South-East Asia, and Western Pacific regions were all estimated to have achieved major improvements in the paediatric CDR. Although this study did not estimate the reasons for these improvements, they coincide with a period of greater attention to tuberculosis control generally (and paediatric tuberculosis specifically) and increasing use of higher-sensitivity tuberculosis diagnostics.^36^ Globally, most unreported cases were in the 0–4 years age group, a group for which tuberculosis disease represents a major mortality risk in the absence of prompt diagnosis and treatment.^10^ Although this study did not estimate paediatric tuberculosis mortality, other studies have estimated high values,^37^ and the high case-fatality rate for untreated paediatric tuberculosis implies that substantial morbidity and mortality is associated with the missed treatment implied by our results.

Although our results showed systematic differences between world regions, we also observed large differences in estimated CDRs between otherwise similar countries in the same region, such as a high estimated CDR for Myanmar compared with low CDRs in Cambodia, Vietnam, and Thailand. There is little evidence to support an explanation for the magnitude of some of these differences as arising from unmodelled differences in tuberculosis epidemiology in these countries. These differences could reflect variation in clinical or programmatic approaches to tuberculosis case detection, broader differences in health system performance, or reporting problems.

Although the incidence estimates from this analysis are substantially greater than reported notifications, they are similar to earlier modelled estimates^1,13–15^ and largely support the magnitude of underreporting estimated by WHO.^1^ Our estimates provide an independent confirmation of the proportion of paediatric tuberculosis cases never diagnosed and reported to WHO, based on a different estimation approach than that in earlier methods. Despite the agreement around aggregate results, there are major differences between our estimates and WHO estimates for individual countries. In general, the magnitude of these differences was proportional to absolute incidence in each country. The discrepancy was particularly large for India, which forms 28% of the global paediatric tuberculosis burden in WHO estimates and 19% in ours. Our estimates were generally greater than incidence estimates reported by IHME, including for India. Given the importance of India as the country with the single largest tuberculosis epidemic globally, further work to understand these differences is warranted.

Compared with earlier modelling approaches,^13–15^ the model developed for this analysis accounted for a greater range of factors known to influence paediatric risk of infection and disease, specifically considering the role of undernutrition in raising tuberculosis risk. The analytical model is most similar to the household model developed by Dodd and colleagues,^14^ which used detailed data from DHS and tuberculosis prevalence surveys to parameterise patterns of exposure. By using recently published respiratory contact matrices and a parametric model for tuberculosis prevalence, we were able to apply our method in countries that did not have DHS and tuberculosis prevalence data available, and thus cover the large majority of countries that contribute to the global tuberculosis burden. The calibration procedure also contributed to the robustness of model estimates by enforcing consistency with reported data for countries where case detection and reporting are thought to be more reliable.

Our analysis had several limitations. First, although we were able to estimate the gap between reported cases and estimated incidence for individual countries, we cannot distinguish what proportion of this gap is due to underdiagnosis and what proportion is due to incomplete reporting. Although both are problematic, the harms implied by these two issues are different, as are the approaches for resolving them. Second, the analysis implicitly ignored potential overdiagnosis (ie, incorrect tuberculosis diagnoses among children with other conditions). Although false-positive paediatric tuberculosis diagnoses are generally understood to be uncommon, they are still possible. If false-positive diagnoses were to represent a non-trivial proportion of total reported cases, it would lead to this analysis underestimating the extent of underreporting in a given country. If false-positive diagnoses were a widespread problem in the countries used for calibration, this would induce a model bias, inflating estimates of incidence and underreporting for all countries. Third, although the analytical model accounted for several of the major mechanisms that create differences in tuberculosis burden between countries, other factors were omitted and the true relationships might not follow the functional forms assumed in the model (such as the linear relationships implied by the risk ratios for HIV, undernutrition, and BCG vaccination). This kind of model misspecification could bias estimates for individual countries and would not be captured by reported credible intervals. Unmodelled differences between the countries used for calibration and other countries could also lead to bias. Fourth, the calibration process required assumptions about levels of underreporting in high-performing countries, for which we assumed that paediatric reporting was almost as high as that achieved for adults in the same country. Given the difficulties of paediatric tuberculosis diagnosis, this assumption could lead to conservative estimates of paediatric tuberculosis incidence. If paediatric underreporting in calibration countries is greater than that assumed in this analysis, then predicted incidence values for other countries will be too low. Finally, we did not account for factors such as HIV that might modify the duration of adult disease and thereby influence the paediatric infectious exposure.

Notwithstanding these limitations, our findings have major policy implications. Tuberculosis in children is treatable. With improved diagnosis of tuberculosis in children, thousands of unnecessary deaths could be avoided. There is an imperative to reinforce surveillance systems to allow more consistent case reporting, strengthen primary health care to ensure prompt access to appropriate diagnosis and care for children developing tuberculosis, expand access to preventive care for children exposed to infection, and invest in research to develop affordable, accurate, and easy-to-use diagnostics for diagnosing tuberculosis in high-burden settings.

## Supplementary Material

1

2

## Figures and Tables

**Table 1:** Global and regional estimates of paediatric tuberculosis incidence for 2019

	0–4 years	5–14 years	Total
Africa	215 300 (180 800–260 500)	213 600 (183 100–251 900)	428 900 (375 900–499 300)
Americas	8400 (7200–9800)	11 387 (9800–13 200)	19 796 (17 500–22 600)
Eastern Mediterranean	50 800 (41 600–62 800)	49 300 (42 200–58 200)	100 100 (86 700–117 600)
Europe	4100 (3500–4700)	7100 (6200–8200)	11 175 (10 000–12 700)
South-East Asia	153 500 (122 800–193 200)	174 500 (149 300–205 200)	328 000 (280 700–387 000)
Western Pacific	48 800 (41 300–58 100)	60 600 (52 200–70 700)	109 400 (96 500–125 00)
Total	481 000 (398 400–587 400)	516 500 (442 900–608 000)	997 500 (868 700–1 163 100)

Data are estimated incidence (equal-tailed 95% credible interval), rounded to three significant digits. Total incidence estimates were calculated as the sum of tuberculosis incidence values estimated for each WHO region and age group.

**Table 2:** Estimates of paediatric (age 0–14 years) tuberculosis incidence and the coverage of case reporting for 30 countries with high tuberculosis burden in 2019

	Incidence estimate (95% CrI)	Reported case notifications	Reported cases as percentage of estimated incidence (95% CrI)
India	193 000 (164 300–228 900)	145 574	75% (64–89)
Nigeria	88 700 (77 400–103 700)	9462	11% (9–12)
Indonesia	70 100 (60 200–82 600)	70 092	100% (85–116)
DR Congo	64 600 (56 400–75 400)	0	0 (0–0)
Pakistan	61 700 (53 100–72 800)	45 447	74% (62–86)
Philippines	58 300 (51 300–67 600)	42 669	73% (63–83)
South Africa	28 800 (25 300–33 600)	16 461	57% (49–65)
Ethiopia	27 100 (23 700–31 600)	11 024	41% (35–47)
Angola	25 600 (22 400–29 800)	8818	34% (30–39)
China	25 000 (21 800–28 700)	6656	27% (23–31)
Tanzania	24 400 (21 300–28 600)	12 240	50% (43–57)
Bangladesh	23 900 (20 400–28 200)	12 330	52% (44–60)
Myanmar	23 700 (20 700–27 500)	23 703	100% (86–114)
Mozambique	20 600 (17 700–24 200)	12 856	62% (53–73)
Kenya	18 700 (16 100–21 900)	8299	44% (38–51)
Zambia	11 000 (9500–12 900)	2473	22% (19–26)
Vietnam	9800 (8500–11 600)	1704	17% (15–20)
Papua New Guinea	6900 (6000–8000)	6859	100% (85–115)
North Korea	6800 (5900–7900)	4626	68% (59–79)
Brazil	5500 (4900–6200)	2681	49% (43–55)
Central African Republic	5200 (4600–6100)	1648	31% (27–36)
Zimbabwe	4800 (4200–5700)	1171	24% (21–28)
Cambodia	4600 (3900–5500)	0	0% (0–0)
Sierra Leone	3700 (3200–4300)	2350	63% (54–73)
Thailand	3500 (3000–4000)	874	25% (22–29)
Congo (Brazzaville)	3400 (3000–4000)	968	28% (24–32)
Liberia	2300 (2000–2700)	1413	61% (53–70)
Russia	2000 (1800–2300)	2028	100% (88–114)
Namibia	1400 (1200–1700)	733	52% (44–60)
Lesotho	1400 (1200–1600)	284	20% (17–24)

Data in parentheses are equal-tailed 95% CrI. Incidence estimates are rounded to the nearest 100. Countries are ordered by estimated incidence. CrI=credible interval.

**Table 3:** Global and regional estimates of paediatric tuberculosis incidence attributable to each of three risk factors in 2019

	HIV	Malnutrition	BCG non-vaccination	Three risk factors combined
Africa	6600 (2500–12 700)	45 100 (19 300–78 100)	71 100 (49 300–97 300)	113 400 (79 900–153 600)
Americas	0 (0–100)	700 (300–1200)	3500 (2600–4600)	4100 (3100–5300)
Eastern Mediterranean	100 (0–100)	14 800 (6300–25 600)	16 000 (11 400–21 600)	28 400 (18 900–40 200)
Europe	0 (0–0)	600 (300–1000)	2800 (2300–3400)	3300 (2700–4000)
South-East Asia	200 (100–500)	56 500 (24 100–97 200)	26 300 (18 700–35 400)	78 400 (45 400–119 600)
Western Pacific	200 (100–400)	9900 (4200–17 000)	14 500 (10 000–19 700)	23 100 (16 000–31 600)
Total	7200 (2700–13 800)	127 600 (54 400–219 700)	134 200 (94 500–181 300)	250 700 (168 000–352 200)

Data are estimated incidence (equal-tailed 95% credible interval). Estimates represent the reduction in incidence produced by the removal of each risk factor.

## Research in context

### Evidence before this study

Childhood tuberculosis is difficult to diagnose and, consequently, substantial underreporting of paediatric tuberculosis cases occurs in many countries. We searched PubMed up to Nov 23, 2021, with no language restrictions for papers reporting methods for estimating the global burden of paediatric tuberculosis, using the search terms “(TB[Title/Abstract] OR tuberc*[Title/Abstract]) AND (incidence[Title/Abstract] OR burden[Title/Abstract]) AND (global[Title/Abstract] or world*[Title/Abstract]) AND (pediatric[Title/Abstract] OR paediatric[Title/Abstract] OR child*[Title/Abstract])”; this search returned 617 entries. Of these, five reported a new approach for estimating paediatric tuberculosis incidence. In each of these approaches, a mathematical model was used to interpret routinely reported data and correct for expected biases due to gaps in case detection. These methods have estimated close to 1 million annual paediatric tuberculosis cases—substantially greater than the total paediatric tuberculosis cases reported by countries to WHO.

### Added value of this study

We developed a novel mathematical model of paediatric tuberculosis incidence that extends the methods developed by earlier analyses and provides new estimates of paediatric tuberculosis incidence, trends in the completeness of case reporting for 185 countries between 2013 and 2019, and estimates of the proportion of cases attributable to specific risk factors. Using this new model, which incorporated several previously unaccounted-for factors known to affect paediatric tuberculosis risk, we estimated that the number of paediatric tuberculosis cases was substantially greater than that reported, with incidence having declined slowly over the study period. Estimated case detection ratios for the paediatric population increased substantially over this period, with improvements concentrated in the Eastern Mediterranean, South-East Asia, and Western Pacific regions. We estimated that a quarter of the current incidence of paediatric tuberculosis can be attributed to elevated disease risks from HIV infection, malnutrition, and BCG non-vaccination.

### Implications of all the available evidence

Studies with different estimation approaches have consistently estimated a large gap in paediatric tuberculosis diagnosis. By use of a novel estimation approach that considers several additional determinants of paediatric tuberculosis incidence, these results further support the large gap in the detection of paediatric tuberculosis cases, and it highlights the effect of modifiable risk factors in increasing total incidence. Urgent action is needed to ensure prompt diagnosis and care for children developing tuberculosis.

## References

[1] WHO. Global tuberculosis report 2020. 2020. https://www.who.int/publications/i/item/9789240013131 (accessed Feb 7, 2021).

[2] Marais BJ, Gie RP, Schaaf HS, The natural history of childhood intra-thoracic tuberculosis: a critical review of literature from the pre-chemotherapy era. Int J Tuberc Lung Dis 2004; 8: 392–402. 15141729

[3] WHO. Roadmap towards ending TB in children and adolescents. Geneva: World Health Organization, 2018.

[4] MacNeil A, Glaziou P, Sismanidis C, Date A, Maloney S, Floyd K. Global epidemiology of tuberculosis and progress toward meeting global targets—worldwide, 2018. MMWR Morb Mortal Wkly Rep 2020; 69: 281–85. 32191687 10.15585/mmwr.mm6911a2 PMC7739980

[5] Hanson C, Osberg M, Brown J, Durham G, Chin DP. Finding the missing patients with tuberculosis: lessons learned from patient-pathway analyses in 5 countries. J Infect Dis 2017; 216 (suppl 7): S686–95. 29117351 10.1093/infdis/jix388 PMC5853970

[6] Roya-Pabon CL, Perez-Velez CM. Tuberculosis exposure, infection and disease in children: a systematic diagnostic approach. Pneumonia 2016; 8: 23. 28702302 10.1186/s41479-016-0023-9 PMC5471717

[7] Kunkel A, Abel Zur Wiesch P, Nathavitharana RR, Marx FM, Jenkins HE, Cohen T. Smear positivity in paediatric and adult tuberculosis: systematic review and meta-analysis. BMC Infect Dis 2016; 16: 282. 27296716 10.1186/s12879-016-1617-9 PMC4906576

[8] Gunasekera KS, Walters E, van der Zalm MM, Development of a treatment-decision algorithm for HIV-uninfected children evaluated for pulmonary tuberculosis. Clin Infect Dis 2021; 73: e904–12. 33449999 10.1093/cid/ciab018 PMC8366829

[9] Marcy O, Borand L, Ung V, A treatment-decision score for HIV-infected children with suspected tuberculosis. Pediatrics 2019; 144: e20182065. 31455612 10.1542/peds.2018-2065

[10] Jenkins HE, Yuen CM, Rodriguez CA, Mortality in children diagnosed with tuberculosis: a systematic review and meta-analysis. Lancet Infect Dis 2017; 17: 285–95. 27964822 10.1016/S1473-3099(16)30474-1 PMC5330933

[11] Allwood BW, Byrne A, Meghji J, Rachow A, van der Zalm MM, Schoch OD. Post-tuberculosis lung disease: clinical review of an under-recognised global challenge. Respiration 2021; 100: 751–63. 33401266 10.1159/000512531

[12] Seddon JA, Jenkins HE, Liu L, Counting children with tuberculosis: why numbers matter. Int J Tuberc Lung Dis 2015; 19 (suppl 1): 9–16. 26564535 10.5588/ijtld.15.0471 PMC4708268

[13] Jenkins HE, Tolman AW, Yuen CM, Incidence of multidrug-resistant tuberculosis disease in children: systematic review and global estimates. Lancet 2014; 383: 1572–79. 24671080 10.1016/S0140-6736(14)60195-1 PMC4094366

[14] Dodd PJ, Gardiner E, Coghlan R, Seddon JA. Burden of childhood tuberculosis in 22 high-burden countries: a mathematical modelling study. Lancet Glob Health 2014; 2: e453–59. 25103518 10.1016/S2214-109X(14)70245-1

[15] Dodd PJ, Sismanidis C, Seddon JA. Global burden of drug-resistant tuberculosis in children: a mathematical modelling study. Lancet Infect Dis 2016; 16: 1193–201. 27342768 10.1016/S1473-3099(16)30132-3

[16] Dodd PJ, Sismanidis C, Glaziou P. Methods for estimating tuberculosis incidence and mortality by age and sex. Int J Epidemiol 2021; 50: 570–77. 33624797 10.1093/ije/dyaa257 PMC8128472

[17] Kyu HH, Maddison ER, Henry NJ, The global burden of tuberculosis: results from the Global Burden of Disease Study 2015. Lancet Infect Dis 2018; 18: 261–84. 29223583 10.1016/S1473-3099(17)30703-X PMC5831985

[18] Lönnroth K, Castro KG, Chakaya JM, Tuberculosis control and elimination 2010–50: cure, care, and social development. Lancet 2010; 375: 1814–29. 20488524 10.1016/S0140-6736(10)60483-7

[19] Prem K, van Zandvoort K, Klepac P, Projecting contact matrices in 177 geographical regions: an update and comparison with empirical data for the COVID-19 era. PLoS Comput Biol 2021; 17: e1009098. 34310590 10.1371/journal.pcbi.1009098 PMC8354454

[20] UN Population Division. World population prospects 2019, online edition, rev 1. 2019. https://population.un.org/wpp/Download/Standard/Population/ (accessed Dec 25, 2020).

[21] WHO. Global tuberculosis programme. 2020. http://www.who.int/tb/country/data/download/en/ (accessed Dec 25, 2020).

[22] Global Burden of Disease Collaborative Network. GBD results tool. 2020. http://ghdx.healthdata.org/gbd-results-tool (accessed Dec 25, 2020).

[23] UNAIDS. AIDS info epidemiological estimates database. https://aidsinfo.unaids.org/ (accessed Feb 6, 2021).

[24] WHO. WHO–UNICEF estimates of BCG coverage. https://apps.who.int/immunization_monitoring/globalsummary/timeseries/tswucoveragebcg.html (accessed Jan 23, 2021).

[25] Jackson CH, Jit M, Sharples LD, De Angelis D. Calibration of complex models through Bayesian evidence synthesis: a demonstration and tutorial. Med Decis Making 2015; 35: 148–61. 23886677 10.1177/0272989X13493143 PMC4847637

[26] Tiemersma EW, van der Werf MJ, Borgdorff MW, Williams BG, Nagelkerke NJD. Natural history of tuberculosis: duration and fatality of untreated pulmonary tuberculosis in HIV negative patients: a systematic review. PLoS One 2011; 6: e17601. 21483732 10.1371/journal.pone.0017601 PMC3070694

[27] Frascella B, Richards AS, Sossen B, Subclinical tuberculosis disease-a review and analysis of prevalence surveys to inform definitions, burden, associations and screening methodology. Clin Infect Dis 2020; 73: e830–41. 10.1093/cid/ciaa1402 PMC8326537 32936877

[28] Sutherland I, Fayers PM. The association of the risk of tuberculous infection with age. Bull Int Union Tuberc 1975; 50: 70–81. 1218289

[29] Styblo K, Meijer J. The quantified increase of the tuberculosis infection rate in a low prevalence country to be expected if the existing MMR programme were discontinued. Bull Int Union Tuberc 1980; 55: 3–8. 7214059

[30] Martinez L, Cords O, Horsburgh CR, The risk of tuberculosis in children after close exposure: a systematic review and individual-participant meta-analysis. Lancet 2020; 395: 973–84. 32199484 10.1016/S0140-6736(20)30166-5 PMC7289654

[31] Dodd PJ, Prendergast AJ, Beecroft C, Kampmann B, Seddon JA. The impact of HIV and antiretroviral therapy on TB risk in children: a systematic review and meta-analysis. Thorax 2017; 72: 559–75. 28115682 10.1136/thoraxjnl-2016-209421 PMC5520282

[32] Lönnroth K, Williams BG, Cegielski P, Dye C. A consistent log-linear relationship between tuberculosis incidence and body mass index. Int J Epidemiol 2010; 39: 149–55. 19820104 10.1093/ije/dyp308

[33] Mangtani P, Abubakar I, Ariti C, Protection by BCG vaccine against tuberculosis: a systematic review of randomized controlled trials. Clin Infect Dis 2014; 58: 470–80. 24336911 10.1093/cid/cit790

[34] Carpenter B, Gelman A, Hoffman MD, Stan: a probabilistic programming language. J Stat Softw 2017; 76. 10.18637/jss.v076.i01 PMC9788645 36568334

[35] Briggs AH, Weinstein MC, Fenwick EAL, Karnon J, Sculpher MJ, Paltiel AD. Model parameter estimation and uncertainty analysis: a report of the ISPOR-SMDM Modeling Good Research Practices Task Force Working Group-6. Med Decis Making 2012; 32: 722–32. 22990087 10.1177/0272989X12458348

[36] Nicol MP, Workman L, Prins M, Accuracy of Xpert MTB/RIF Ultra for the diagnosis of pulmonary tuberculosis in children. Pediatr Infect Dis J 2018; 37: e261–63. 29474257 10.1097/INF.0000000000001960

[37] Dodd PJ, Yuen CM, Sismanidis C, Seddon JA, Jenkins HE. The global burden of tuberculosis mortality in children: a mathematical modelling study. Lancet Glob Health 2017; 5: e898–906. 28807188 10.1016/S2214-109X(17)30289-9 PMC5556253

