# Supplementary material for: Global estimates of paediatric tuberculosis incidence in 2013–19: a mathematical modelling analysis
Source: Lancet Glob Health. Author manuscript; Available in PMC 2022 Feb 1. (PMC8800006; doi:10.1016/S2214-109X(21)00462-9)
Supplement: 1 [file NIHMS1772784-supplement-1.pdf]

THE LANCET
Global Health

Supplementary appendix

This appendix formed part of the original submission and has been peer reviewed. We post it as supplied by the authors.

Supplement to: Yerramsetti S, Cohen T, Atun R, Menzies NA. Global estimates of paediatric tuberculosis incidence in 2013–19: a mathematical modelling analysis. *Lancet Glob Health* 2021; published online Dec 8. https://doi.org/10.1016/S2214-109X(21)00462-9.

Mathematical model of pediatric TB exposure and natural history

In the mathematical model, the pediatric population is stratified by country (185 strata), year (7 strata: 2013 to 2019), and age group (2 strata: 0-4 years, 5-14 years). The absolute number of incident pediatric TB cases for a given stratum (N_{jkt} for age group *j* in country *k* and year *t*) is calculated as the product of population size (P_{jkt}) and the incidence rate for that group. The incidence rate is calculated as a function of the number of effective infectious exposures per child (l_{jkt}), the probability of TB disease conferred by *Mtb* infection (a_j), and the prevalence of conditions (h_{jkt} for untreated HIV, t_{jkt} for treated HIV, u_{jkt} for underweight, and v_{jkt} for vaccination with BCG) that increase or reduce the risk of disease progression. Table S5A-B shows country-specific input values for 2019. Based on these inputs, the number of incident pediatric TB cases in age group *j* in country *k* and year *t* is given by equation 1:

$$N_{jkt} = P_{jkt} l_{jkt} \left(1 - (1 - m^h) h_{jkt} - (1 - m^h m^t) t_{jkt}\right)\left(1 - (1 - m^u) u_{jkt}\right)\left(1 - (1 - m_k^v) v_{jkt}\right) a_j \quad [1]$$

In this equation, m^h, m^u, and m_k^v represent risk-ratios for progression to TB for untreated HIV, underweight, and BCG vaccination respectively. m^t is the risk-ratio for progression for treated vs. untreated HIV. We estimated the annual number of effective infectious exposures per child (l_{jkt}) as the exposure to transmission from infectious adults, based on evidence that adults represent the large majority of source cases for pediatric *Mtb* infection (1). To do so, we calculated the number of infectious contacts as a function of the number of respiratory contacts by pediatric individuals with each adult age group (c_{ijk}, for the number of contacts for a child in age group *j* with adults in age group *i* in country *k*), and TB disease prevalence in each adult age group. WHO TB burden estimates do not include age-stratified estimates of TB disease prevalence, and instead

we estimated adult TB prevalence by multiplying adult TB incidence rates by country, year, and age group (r_{ikt}) by the average duration of TB disease (d_{kt}). This relies on the assumption that, unless incidence rates are changing rapidly, prevalence is related to incidence by the relationship prevalence = incidence*duration.

For each country and year, we estimated d_{kt} by assuming that individuals developing TB disease exit the disease state to either self-cure, death, or treatment. We modeled the infectious period as an initial subclinical stage (2) where exit rates are zero, followed by a clinical disease stage where the three exit rates are applied in a competing risks framework. We assumed subclinical and clinical stages were equally infectious. This model was parameterized as the total duration of infectiousness without treatment (d_{untx}), the fraction (f) of this total represented by the subclinical stage, and the fraction of all incident TB cases that are treated (s_{kt} for country *k* and year *t*). With these assumptions, d_{kt} can be estimated for each country and year:

$$d_{kt} = d_{untx}\big(1 - s_{kt}(1 - f)\big) \qquad [2]$$

With random mixing, the average annual number of effective infectious exposures (l_{jkt}) for a given stratum can be calculated by summing $c_{ijk}r_{ikt}d_{kt}$ across adult age groups (i). However, this does not account for contact saturation (3), which will produce a sublinear relationship between l_{jkt} and both c_{ijk} and d_{kt}. For this reason, we specified equation 3 to calculate the number of effective contacts, in which the additional parameter q determines the extent of contact saturation. This contact saturation parameter relaxes the assumption that transmission increases linearly in the average number of infectious contacts for each adult TB case (where the average number of contacts per case is the product of the duration of disease and average annual contacts). With the formulation

shown in Equation 3, transmission increases monotonically with the average number of contacts per case, with a positive first derivative and negative second derivative.

$$l_{jkt} = \frac{\sum_i c_{ijk} r_{ikt}}{\sum_i c_{ijk}} \left[\frac{1 - \exp(-b d_{kt} q \sum_i c_{ijk})}{q} \right] \quad [3]$$

<u>Model calibration</u>

Twenty-seven countries met the criteria used for defining better pediatric case detection (WHO-reported CDR ≥ 0.85 averaged over the study period, and age-standardized death-to-notification ratios in the lowest quintile of countries over the study period): Albania, Austria, Belgium, Brazil, Cuba, Germany, Israel, Japan, Kazakhstan, Kuwait, Malaysia, New Zealand, Oman, Poland, Portugal, South Korea, Moldova, Romania, Serbia, Singapore, Slovakia, Spain, Switzerland, Turkey, United Kingdom, USA, and Uruguay.

We specified a negative binomial likelihood function for pediatric case notifications in each of the 27 countries. For each country we assumed that the WHO-reported case detection ratio applied, multiplied by a parameter g representing the relative probability of case detection for pediatric vs. adult cases, in countries used for model calibration. As a consequence, the mean of the negative binomial likelihood was calculated as the product of N_{jkt}, s_{kt}, and g. We used a Half-Normal(0, 1) prior for the inverse square-root of the negative binomial dispersion parameter (ϕ) (4).

The calibration was implemented with Stan (5, 6), which generates samples from the posterior distribution of a probability model using a Hamiltonian Monte Carlo algorithm (7). We ran the sampler with 4 chains of 11,000 iterations each. We discarded the first 10,000 draws as warm-up, and retained the final 1,000 from each chain, producing a

posterior sample of 4,000 parameter sets. Convergence diagnostics suggested that sampling was successful: the maximum Rhat value was <1.001, the minimum effective sample size was 1795, and there were no divergent transitions or other error messages reported.

Figure S1: Schematic of natural history model for pediatric TB, and evidence sources used for model parametrization and calibration.*

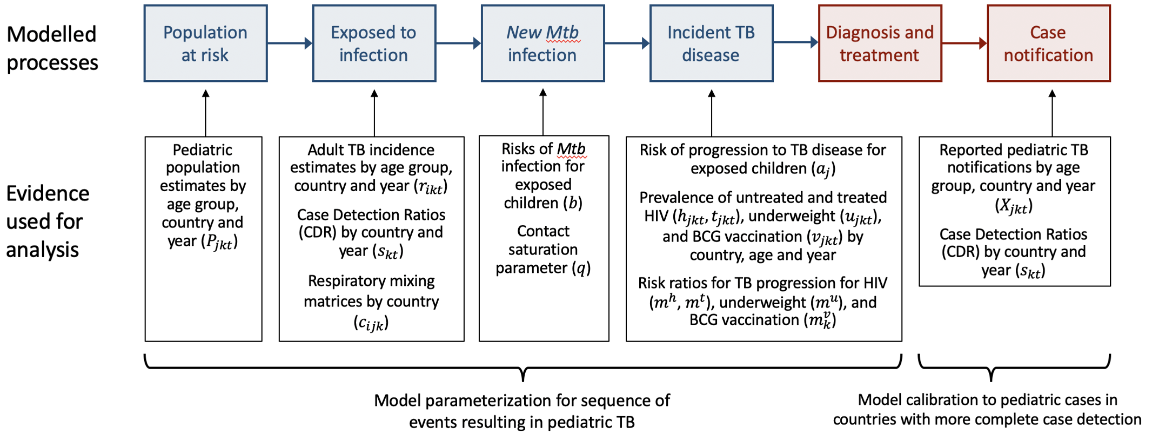

* Subscripts represent adult age group (*i*), pediatric age group (*j*), country (*k*), and calendar year (*t*).

<u>Table S1: Model parameters and input data.</u>

Name	Description	Value (mean [95% interval])	Source
P_{jkt}	Population size for each pediatric stratum	Fixed value for each age group *j*, country *k*, and year *t*	United Nations Population Division (8)
s_{kt}	Fraction of all adult TB cases treated	Fixed value for each country *k* in year *t*	WHO TB Programme (9)
c_{ijk}	Average number of respiratory contacts per year for children	Fixed value for each pediatric age group *j* with adult age group *i* and country *k*	Prem 2020 (10)
r_{ikt}	TB incidence rate among adults	Fixed value for each adult age group *i*, in country *k*, and year *t*	WHO TB Programme (9)
h_{jkt}	Prevalence of untreated HIV in children	Fixed value for age group *j*, country *k*, and year *t*	GBD Collaborative Network (11), UNAIDS (12)
t_{jkt}	Prevalence of treated HIV in children	Fixed value for age group *j*, country *k*, and year *t*	GBD Collaborative Network (11), UNAIDS (12)
u_{jkt}	Prevalence of protein-energy malnutrition (moderate or severe acute wasting) in children	Fixed value for age group *j*, country *k*, and year *t*	Global Burden of Disease Collaborative Network (11)
v_{jkt}	Coverage of BCG vaccination	Fixed value for age group *j*, country *k*, and year *t*	WHO-UNICEF (13)
X_{jkt}	Reported pediatric notifications in countries used for calibration	Fixed value for each age group *j*, country *k*, and year *t*	WHO TB Programme (9)
d_{untx}	Average duration of TB disease without treatment (years)	3.0 [2.5, 3.5]<br>Distribution: Gamma(138, 46)	Tiemersma 2011 (14)
f	Fraction of total duration of disease represented by subclinical TB	0.25 [0.17, 0.33]<br>Distribution: Beta(28, 83)	Frascella 2020 (2)
q	Rate of contact saturation for infectious disease cases	0.50 [0.09, 0.91]<br>Distribution: Beta(2, 2)	Estimated through calibration
b	Per-contact transmission probability for children exposed to infectious adults	0.0047 [0.0023, 0.0058]<br>Distribution: Beta(27, 5770)	Sutherland 1975, Styblo 1980 (15, 16)
a_j	Probability of TB disease following infectious exposure, by age group	*0-4 years old*: 0.19 [0.08, 0.37]<br>Distribution: Beta(5, 21)<br><br>*5-14 years old*: 0.09 [0.05, 0.16]<br>Distribution: Beta(9, 92)	Martinez 2020 (17)
m^h	Risk ratio for progression to TB disease following infectious exposure, with untreated HIV	7.9 [4.5, 13.7]<br>Distribution: Gamma(11, 1)	Dodd 2017 (18)
m^t	Risk ratio for progression to TB disease following infectious exposure, treated HIV vs. untreated HIV	0.30 [0.21, 0.39]<br>Distribution: Gamma(43, 142)	Dodd 2017 (18)
m^u	Risk ratio for TB disease following infectious exposure, with underweight	4.0 [2.0, 6.0]<br>Distribution: Gamma(15, 4)	Lönnroth 2010 (19)
m_k^v	Risk ratio for TB disease following infectious exposure, with BCG vaccination	*Latitude >40°*: 0.31 [0.20, 0.45]<br>Distribution: Gamma(23, 74)<br><br>*Latitude 20-40°*: 0.68 [0.43, 0.99]<br>Distribution: Gamma(23, 34)<br><br>*Latitude <20°*: 0.77 [0.49, 1.12]<br>Distribution: Gamma(23, 30)<br><br>These three parameters were assumed to be perfectly correlated.	Mangtani 2014 (20)
g	Relative probability of case detection for pediatric vs. adult cases, in countries used for model calibration	0.90 [0.80, 0.95]<br>Distribution: Beta(53, 6)	Assumption

Table S2: Posterior distribution for model parameters.

Parameter definition		Parameter values			
Name	Description	Mean	Standard deviation	2.5th percentile	97.5th percentile
d_{untx}	Average duration of TB disease without treatment (years)	3.05	0.25	2.58	3.54
f	Fraction of total duration of disease represented by subclinical TB	0.249	0.042	0.172	0.337
q	Rate of contact saturation for infectious disease cases	0.403	0.086	0.254	0.584
b	Per-contact transmission probability for children exposed to infectious adults	0.00500	0.00084	0.00356	0.00682
a_j	Probability of TB disease following infectious exposure, by age group	*0-4 years old:* 0.172	0.034	0.112	0.242
		5-14 years old: 0.126	0.024	0.085	0.175
m^h	Risk ratio for progression to TB disease following infectious exposure, with untreated HIV	7.92	2.39	3.95	13.25
m^t	Risk ratio for progression to TB disease following infectious exposure, treated HIV vs. untreated HIV	0.301	0.047	0.217	0.398
m^u	Risk ratio for TB disease following infectious exposure, with underweight	3.96	1.03	2.22	6.28
m_k^v	Risk ratio for TB disease following infectious exposure, with BCG vaccination	*Latitude >40°:* 0.208	0.019	0.172	0.248
		Latitude 20-40°: 0.456	0.042	0.378	0.543
		Latitude <20°: 0.516	0.048	0.428	0.615
g	Relative probability of case detection for pediatric vs. adult cases, in countries used for model calibration	0.906	0.036	0.829	0.964
ϕ	Negative binomial dispersion parameter	1.7	0.13	1.46	1.96

Figure S2: Scatterplots of posterior distribution of model parameters.*

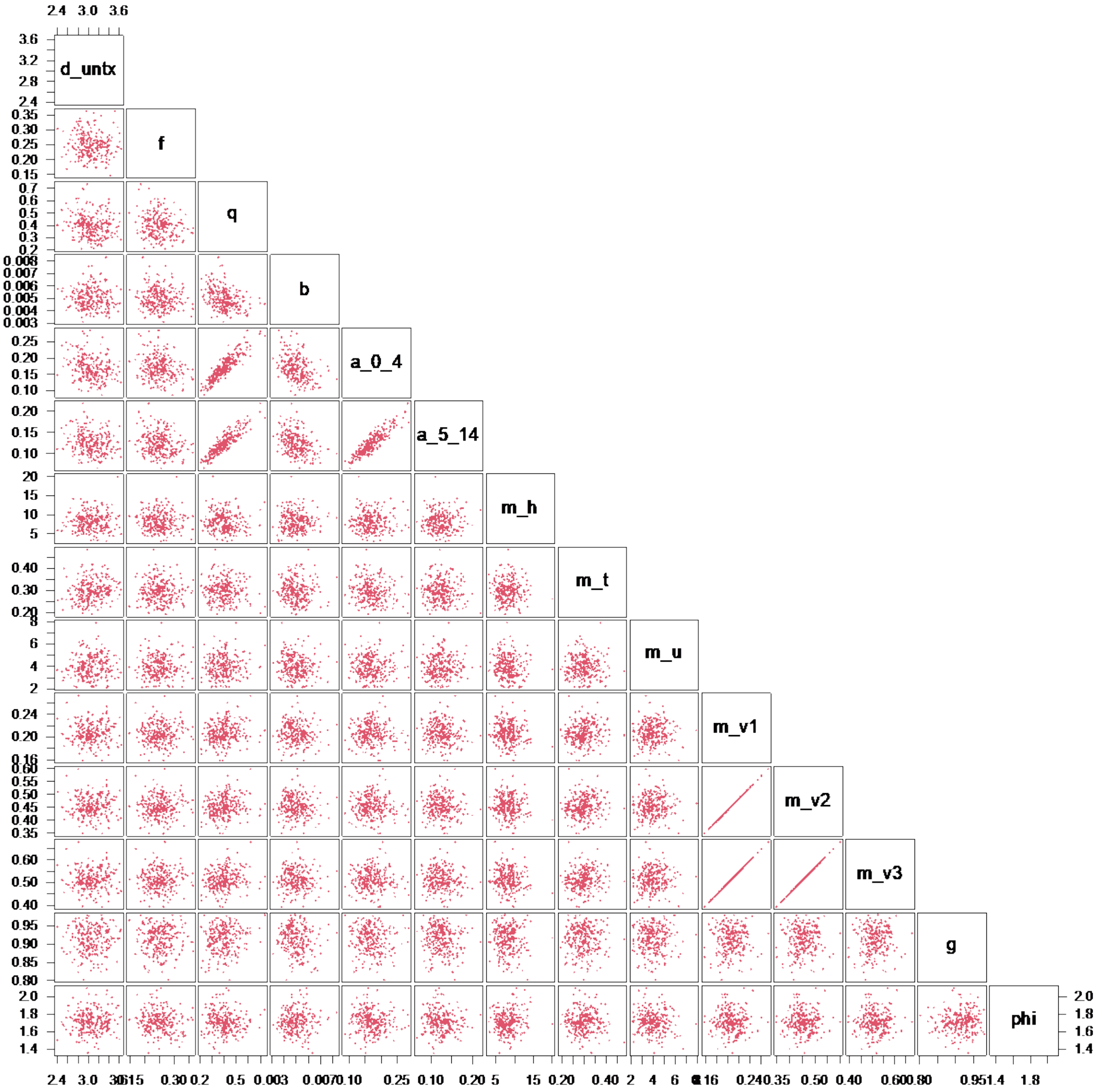

* Scatterplots based on a random sample of 200 parameters sets. Most parameters had low correlation with other parameters. Exceptions were q and a_{0-4} (Spearman rank correlation coefficient (rho) = 0.89), a_{0-4} and a_{5-14} (rho = 0.89), q and a_{5-14} (rho = 0.88) , b and a_{5-14} (rho = -0.28), b and a_{0-4} (rho = -0.27), b and q (rho = -0.22). All three m_k^v parameters are perfectly correlated by construction. All other parameters have rank correlation within [-02, 0.2].

Figure S3: Fitted values compared to reported case notifications in 2018 for twenty-seven countries used for model calibration.*

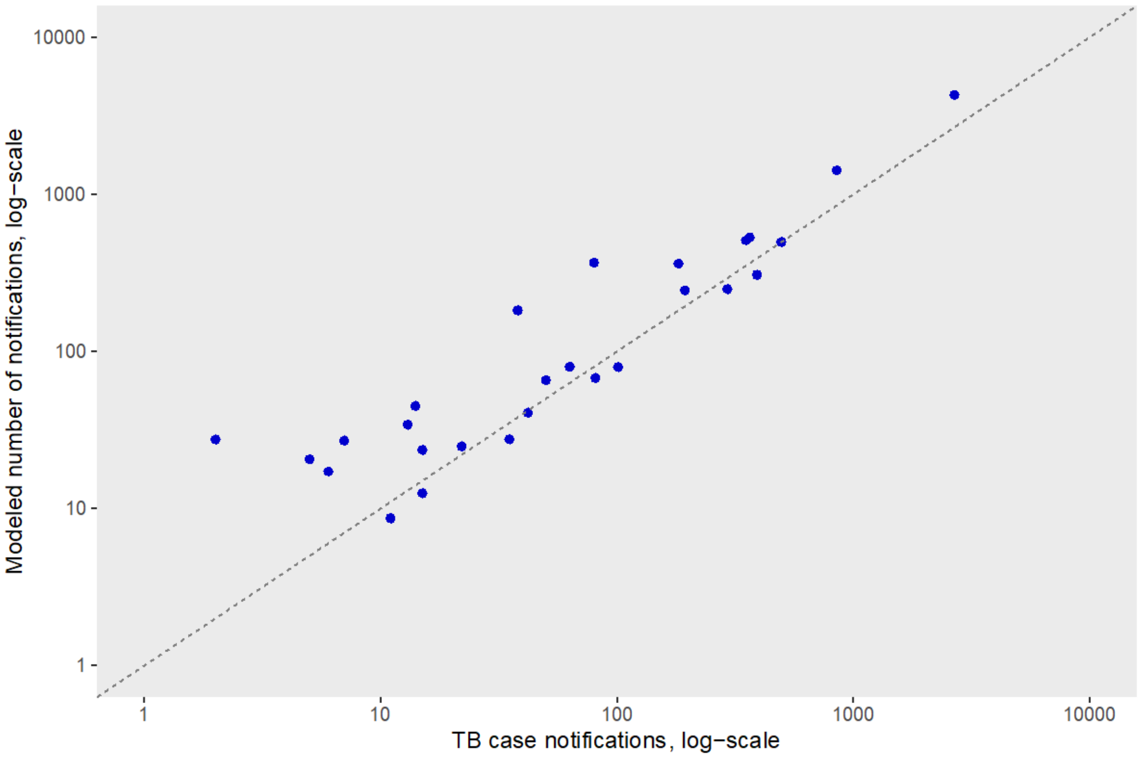

* Countries included: Albania, Austria, Belgium, Brazil, Cuba, Germany, Israel, Japan, Kazakhstan, Kuwait, Malaysia, New Zealand, Oman, Poland, Portugal, South Korea, Moldova, Romania, Serbia, Singapore, Slovakia, Spain, Switzerland, Turkey, United Kingdom, USA, and Uruguay.

Table S3: Estimates of pediatric TB incidence and case detection ratio (CDR) for all countries in 2019.

Country	Incidence estimate	Reported case notifications	Reported cases as percentage of estimated incidence (%)
Afghanistan	11,598 (10,180 - 13,481)	11474	99 (85 - 113)
Albania	11 (10 - 13)	11	100 (87 - 114)
Algeria	1,905 (1,638 - 2,239)	0	0 (0 - 0)
Angola	25,584 (22,441 - 29,775)	8818	34 (30 - 39)
Antigua and Barbuda	0 (0 - 0)	0	---
Argentina	897 (784 - 1,032)	897	100 (87 - 114)
Armenia	38 (33 - 44)	38	100 (86 - 116)
Australia	154 (131 - 181)	0	0 (0 - 0)
Austria	33 (28 - 38)	22	66 (57 - 77)
Azerbaijan	302 (263 - 350)	179	59 (51 - 68)
Bahamas	7 (6 - 9)	6	82 (70 - 95)
Bahrain	17 (14 - 20)	4	24 (20 - 28)
Bangladesh	23,932 (20,412 - 28,199)	12330	52 (44 - 60)
Barbados	0 (0 - 0)	0	---
Belarus	46 (40 - 52)	8	18 (15 - 20)
Belgium	83 (71 - 97)	50	60 (51 - 70)
Belize	10 (8 - 11)	0	0 (0 - 0)
Benin	1,162 (1,011 - 1,361)	231	20 (17 - 23)
Bhutan	73 (64 - 85)	28	38 (33 - 44)
Bolivia	1,061 (933 - 1,235)	245	23 (20 - 26)
Bosnia and Herzegovina	11 (9 - 12)	3	28 (25 - 32)
Botswana	518 (447 - 610)	0	0 (0 - 0)
Brazil	5,456 (4,857 - 6,179)	2681	49 (43 - 55)
Brunei Darussalam	14 (12 - 17)	6	42 (36 - 48)
Bulgaria	54 (48 - 62)	54	100 (88 - 114)
Burkina Faso	1,889 (1,608 - 2,252)	133	7 (6 - 8)
Burundi	2,476 (2,147 - 2,902)	324	13 (11 - 15)
Cote d'Ivoire	5,916 (5,124 - 6,944)	1018	17 (15 - 20)
Cabo Verde	20 (17 - 23)	4	20 (17 - 23)
Cambodia	4,619 (3,949 - 5,457)	0	0 (0 - 0)
Cameroon	8,497 (7,472 - 9,900)	1255	15 (13 - 17)
Canada	125 (107 - 145)	0	0 (0 - 0)
Central African Republic	5,237 (4,595 - 6,101)	1648	31 (27 - 36)
Chad	5,954 (5,145 - 7,029)	1043	18 (15 - 20)
Chile	122 (106 - 141)	56	46 (40 - 53)
China	24,973 (21,776 - 28,746)	6656	27 (23 - 31)

Country	Incidence estimate	Reported case notifications	Reported cases as percentage of estimated incidence (%)
Colombia	965 (846 - 1,111)	0	0 (0 - 0)
Comoros	46 (40 - 55)	0	0 (0 - 0)
Congo	3,437 (3,024 - 4,009)	968	28 (24 - 32)
Costa Rica	26 (23 - 30)	14	54 (47 - 62)
Croatia	3 (3 - 4)	1	32 (28 - 37)
Cuba	22 (19 - 25)	6	27 (24 - 31)
Cyprus	5 (4 - 6)	2	40 (34 - 47)
Czechia	31 (27 - 36)	4	13 (11 - 15)
North Korea	6,757 (5,854 - 7,875)	4626	68 (59 - 79)
Democratic Republic of the Congo	64,564 (56,371 - 75,442)	0	0 (0 - 0)
Denmark	22 (19 - 26)	16	73 (63 - 85)
Djibouti	197 (167 - 232)	0	0 (0 - 0)
Dominican Republic	321 (277 - 375)	80	25 (21 - 29)
Ecuador	612 (538 - 707)	187	31 (26 - 35)
Egypt	1,027 (891 - 1,205)	488	48 (41 - 55)
El Salvador	296 (261 - 340)	135	46 (40 - 52)
Equatorial Guinea	362 (316 - 423)	96	26 (23 - 30)
Eritrea	451 (386 - 534)	230	51 (43 - 60)
Estonia	3 (2 - 3)	1	37 (33 - 42)
Eswatini	474 (406 - 563)	176	37 (31 - 43)
Ethiopia	27,128 (23,679 - 31,624)	11024	41 (35 - 47)
Fiji	123 (106 - 144)	123	100 (85 - 116)
Finland	15 (13 - 17)	9	62 (53 - 71)
France	459 (392 - 538)	217	47 (40 - 55)
Gabon	1,361 (1,183 - 1,595)	367	27 (23 - 31)
Gambia	743 (645 - 871)	130	18 (15 - 20)
Georgia	89 (78 - 103)	72	81 (70 - 92)
Germany	285 (242 - 332)	194	68 (58 - 80)
Ghana	5,433 (4,686 - 6,391)	823	15 (13 - 18)
Greece	23 (20 - 27)	16	68 (58 - 80)
Grenada	0 (0 - 0)	0	0 (0 - 0)
Guam	13 (11 - 15)	10	78 (66 - 91)
Guatemala	466 (409 - 540)	442	95 (82 - 108)
Guinea-Bissau	1,209 (1,056 - 1,412)	119	10 (8 - 11)
Guinea	4,760 (4,178 - 5,528)	1157	24 (21 - 28)
Guyana	50 (43 - 59)	15	30 (26 - 35)
Haiti	2,085 (1,839 - 2,405)	1340	64 (56 - 73)
Honduras	285 (250 - 331)	49	17 (15 - 20)

Country	Incidence estimate	Reported case notifications	Reported cases as percentage of estimated incidence (%)
Hungary	10 (9 - 11)	10	100 (87 - 115)
Iceland	1 (1 - 2)	0	0 (0 - 0)
India	193,027 (164,279 - 228,894)	145574	75 (64 - 89)
Indonesia	70,092 (60,235 - 82,642)	70092	100 (85 - 116)
Iran	554 (478 - 646)	319	58 (49 - 67)
Iraq	1,883 (1,618 - 2,222)	567	30 (26 - 35)
Ireland	26 (22 - 31)	10	38 (32 - 45)
Israel	34 (29 - 41)	7	20 (17 - 24)
Italy	259 (220 - 306)	172	66 (56 - 78)
Jamaica	18 (16 - 21)	18	100 (86 - 115)
Japan	232 (201 - 269)	38	16 (14 - 19)
Jordan	55 (49 - 64)	29	53 (45 - 60)
Kazakhstan	575 (514 - 651)	351	61 (54 - 68)
Kenya	18,651 (16,123 - 21,885)	8299	44 (38 - 51)
Kiribati	83 (73 - 96)	75	90 (78 - 103)
Kuwait	43 (38 - 50)	13	30 (26 - 34)
Kyrgyzstan	369 (322 - 426)	305	83 (72 - 95)
Lao	1,318 (1,154 - 1,536)	95	7 (6 - 8)
Latvia	9 (8 - 10)	0	0 (0 - 0)
Lebanon	111 (95 - 132)	54	48 (41 - 57)
Lesotho	1,390 (1,188 - 1,647)	284	20 (17 - 24)
Liberia	2,299 (2,017 - 2,679)	1413	61 (53 - 70)
Libya	329 (289 - 381)	103	31 (27 - 36)
Lithuania	28 (25 - 32)	27	95 (83 - 108)
Luxembourg	4 (4 - 5)	0	0 (0 - 0)
Madagascar	11,488 (10,023 - 13,418)	3046	27 (23 - 30)
Malawi	5,027 (4,371 - 5,884)	1527	30 (26 - 35)
Malaysia	1,810 (1,556 - 2,114)	850	47 (40 - 55)
Maldives	11 (9 - 13)	3	27 (22 - 32)
Mali	2,614 (2,268 - 3,067)	343	13 (11 - 15)
Malta	4 (4 - 5)	2	45 (39 - 54)
Mauritania	566 (491 - 666)	156	28 (23 - 32)
Mauritius	6 (5 - 7)	3	50 (43 - 58)
Mexico	2,125 (1,880 - 2,439)	661	31 (27 - 35)
Micronesia	21 (19 - 24)	21	100 (87 - 113)
Mongolia	485 (417 - 571)	406	84 (71 - 97)
Montenegro	2 (2 - 2)	0	0 (0 - 0)
Morocco	2,557 (2,213 - 2,970)	2089	82 (70 - 94)

Country	Incidence estimate	Reported case notifications	Reported cases as percentage of estimated incidence (%)
Mozambique	20,576 (17,693 - 24,237)	12856	62 (53 - 73)
Myanmar	23,703 (20,718 - 27,460)	23703	100 (86 - 114)
Namibia	1,415 (1,222 - 1,660)	733	52 (44 - 60)
Nepal	5,196 (4,489 - 6,106)	1723	33 (28 - 38)
Netherlands	60 (52 - 70)	46	76 (66 - 89)
New Zealand	35 (30 - 41)	2	6 (5 - 7)
Nicaragua	207 (179 - 242)	0	0 (0 - 0)
Niger	6,229 (5,374 - 7,367)	529	8 (7 - 10)
Nigeria	88,739 (77,391 - 103,749)	9462	11 (9 - 12)
North Macedonia	12 (11 - 14)	12	100 (88 - 113)
Norway	16 (13 - 18)	4	26 (22 - 30)
Oman	26 (23 - 30)	5	19 (16 - 22)
Pakistan	61,745 (53,137 - 72,809)	45447	74 (62 - 86)
Panama	113 (98 - 132)	87	77 (66 - 89)
Papua New Guinea	6,859 (5,952 - 8,027)	6859	100 (85 - 115)
Paraguay	236 (208 - 272)	197	83 (72 - 95)
Peru	2,581 (2,274 - 2,972)	1363	53 (46 - 60)
Philippines	58,308 (51,255 - 67,577)	42669	73 (63 - 83)
Poland	86 (76 - 97)	81	94 (83 - 106)
Portugal	101 (87 - 117)	63	62 (54 - 72)
Puerto Rico	2 (2 - 2)	0	0 (0 - 0)
Qatar	24 (21 - 28)	3	12 (11 - 14)
South Korea	432 (366 - 504)	80	19 (16 - 22)
Moldova	101 (90 - 114)	101	100 (88 - 113)
Romania	391 (346 - 444)	391	100 (88 - 113)
Russia	2,028 (1,774 - 2,316)	2028	100 (88 - 114)
Rwanda	963 (830 - 1,132)	434	45 (38 - 52)
Saint Lucia	0 (0 - 0)	0	0 (0 - 0)
Saint Vincent and the Grenadines	0 (0 - 0)	0	0 (0 - 0)
Samoa	2 (2 - 3)	0	0 (0 - 0)
Sao Tome and Principe	41 (36 - 48)	11	27 (23 - 31)
Saudi Arabia	302 (266 - 347)	106	35 (31 - 40)
Senegal	2,956 (2,534 - 3,496)	592	20 (17 - 23)
Serbia	16 (14 - 18)	15	94 (82 - 107)
Seychelles	1 (1 - 1)	0	0 (0 - 0)
Sierra Leone	3,707 (3,241 - 4,316)	2350	63 (54 - 73)
Singapore	57 (49 - 66)	14	25 (21 - 28)

Country	Incidence estimate	Reported case notifications	Reported cases as percentage of estimated incidence (%)
Slovakia	35 (30 - 41)	35	100 (86 - 116)
Slovenia	5 (4 - 6)	2	41 (35 - 47)
Solomon Islands	65 (57 - 75)	53	82 (71 - 93)
Somalia	11,657 (9,874 - 13,900)	3460	30 (25 - 35)
South Africa	28,816 (25,279 - 33,610)	16461	57 (49 - 65)
South Sudan	5,311 (4,554 - 6,278)	3346	63 (53 - 73)
Spain	293 (252 - 340)	293	100 (86 - 116)
Sri Lanka	818 (684 - 985)	237	29 (24 - 35)
Sudan	4,769 (4,044 - 5,711)	1951	41 (34 - 48)
Suriname	22 (19 - 26)	6	27 (23 - 32)
Sweden	40 (34 - 46)	19	48 (41 - 56)
Switzerland	30 (26 - 35)	15	50 (43 - 58)
Syria	294 (256 - 341)	225	77 (66 - 88)
Tajikistan	803 (690 - 946)	404	50 (43 - 59)
Thailand	3,503 (3,049 - 4,029)	874	25 (22 - 29)
Timor-Leste	911 (767 - 1,096)	364	40 (33 - 47)
Togo	472 (407 - 554)	85	18 (15 - 21)
Tonga	1 (1 - 2)	1	70 (60 - 81)
Trinidad and Tobago	22 (19 - 26)	0	0 (0 - 0)
Tunisia	275 (241 - 318)	213	77 (67 - 89)
Turkey	631 (549 - 727)	496	79 (68 - 90)
Turkmenistan	206 (178 - 241)	0	0 (0 - 0)
Uganda	18,278 (15,990 - 21,337)	8218	45 (39 - 51)
Ukraine	847 (754 - 960)	585	69 (61 - 78)
United Arab Emirates	6 (5 - 7)	4	64 (56 - 73)
United Kingdom	449 (387 - 520)	182	41 (35 - 47)
Tanzania	24,443 (21,302 - 28,617)	12240	50 (43 - 57)
United States of America	676 (578 - 792)	364	54 (46 - 63)
Uruguay	52 (45 - 60)	42	81 (70 - 93)
Uzbekistan	2,190 (1,880 - 2,585)	2190	100 (85 - 117)
Vanuatu	17 (15 - 20)	14	83 (71 - 96)
Venezuela	935 (817 - 1,082)	565	60 (52 - 69)
Viet Nam	9,825 (8,452 - 11,550)	1704	17 (15 - 20)
Yemen	2,602 (2,248 - 3,064)	1239	48 (40 - 55)
Zambia	11,000 (9,504 - 12,929)	2473	22 (19 - 26)
Zimbabwe	4,848 (4,179 - 5,696)	1171	24 (21 - 28)

Figure S4: Estimated pediatric TB incidence rate (per 100,000) by country, 2019.

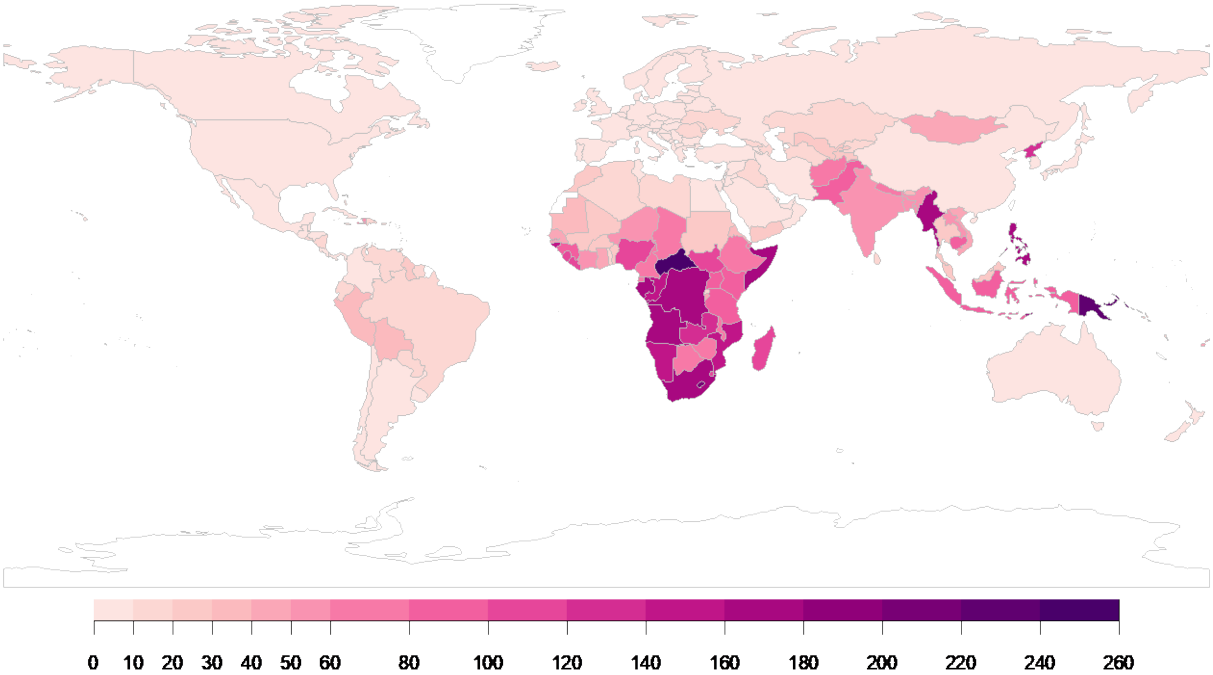

Figure S5: Pediatric TB incidence estimates from this analysis compared to WHO and IHME estimates for 2019.*

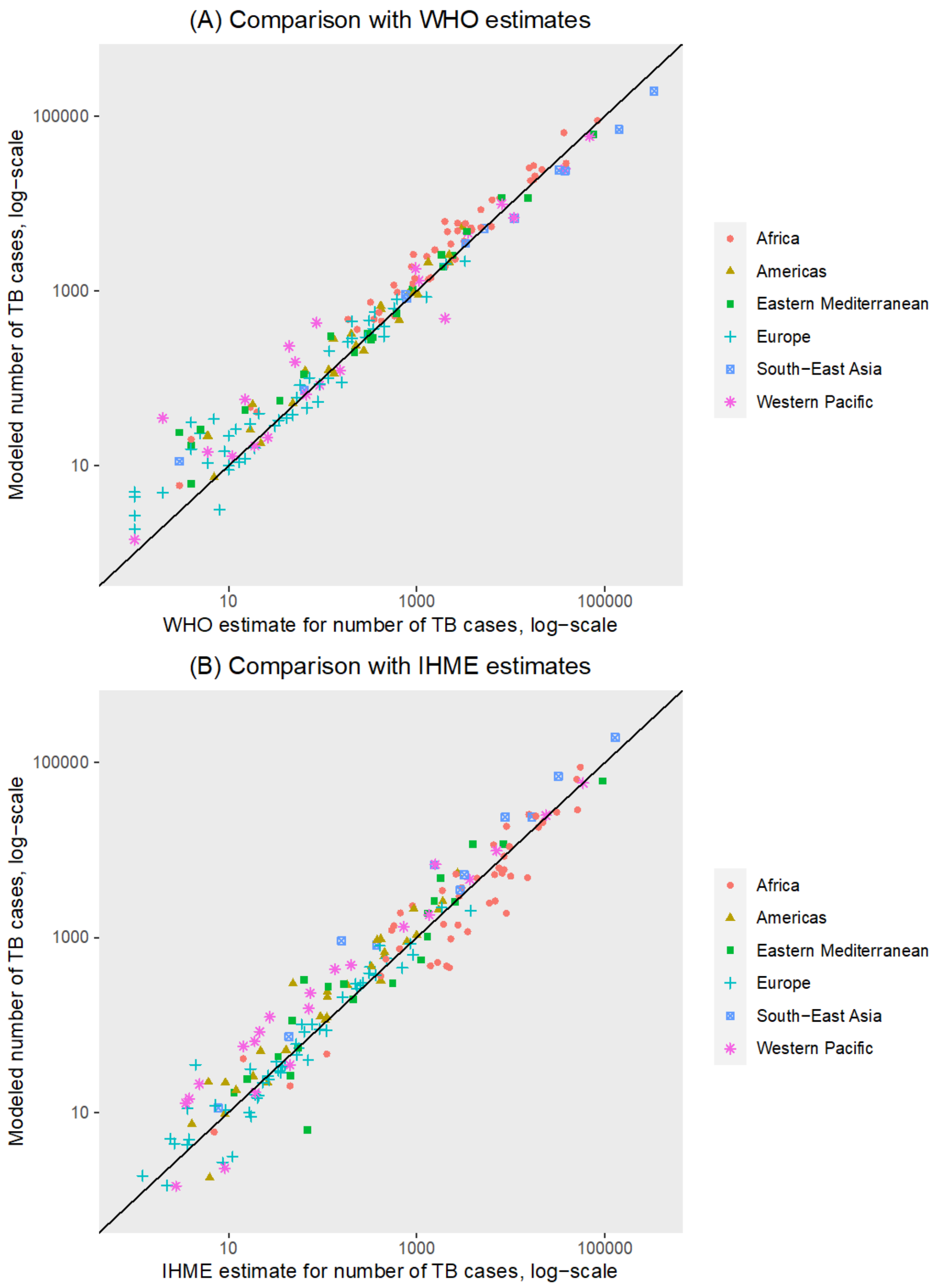

* Panel A: Pediatric TB incidence estimates from this analysis compared to WHO estimates for 2019, for 174 countries (11 countries with <1 estimated pediatric case excluded from plot). Panel B: Pediatric TB incidence estimates from this analysis compared to IHME estimates for 2019, for 179 countries (6 countries with <1 estimated pediatric case excluded from plot)

Figure S6: Model pediatric TB incidence estimates vs. WHO and IHME estimates for 2019, for the thirty WHO high TB burden countries.

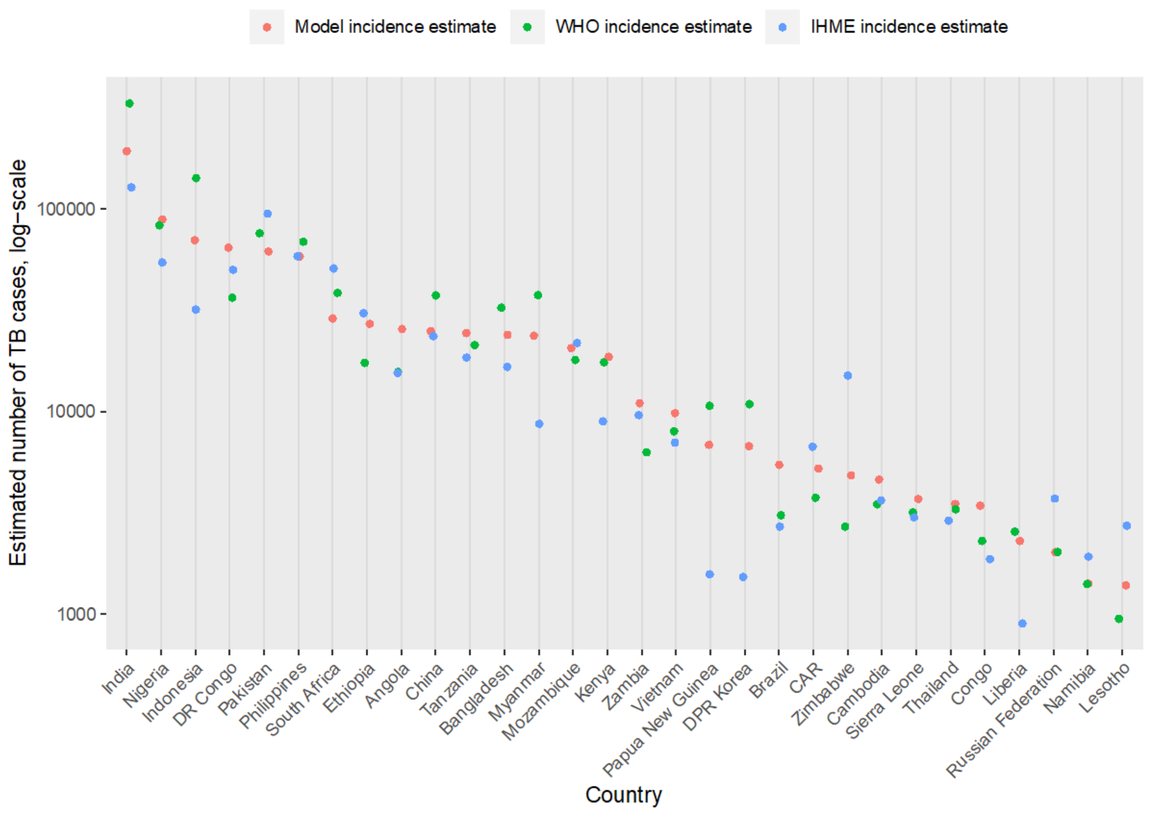

Table S4: Global and regional estimates of the percentage of pediatric TB incidence attributable to each of three risk factors in 2019.*

WHO Region	HIV	Malnutrition	BCG non-vaccination	Three risk factors combined
Africa	1.5% (0.6–2.8)	10.5% (4.7–17.1)	16.6% (11.7–21.8)	26.4% (19.6–33.6)
Americas	0.2% (0.1–0.3)	3.5% (1.5–6.0)	17.8% (13.0–22.9)	20.8% (15.8–26.2)
Eastern Mediterranean	0.1% (0.0–0.1)	14.7% (6.8–23.4)	16.0% (11.5–20.8)	28.3% (20.4–36.9)
Europe	0.0% (0.0–0.1)	5.3% (2.3–9.0)	25.3% (21.0–29.8)	29.5% (24.6–34.6)
South-East Asia	0.1% (0.0–0.1)	17.0% (8.1–26.8)	8.0% (5.7–10.7)	23.7% (15.0–33.2)
Western Pacific	0.2% (0.1–0.3)	9.0% (4.0–14.8)	13.2% (9.2–17.7)	21.1% (15.1–27.7)
TOTAL	0.7% (0.3–1.3)	12.7% (5.8–20.5)	13.5% (9.5–17.8)	25.1% (17.8–33.0)

* Values in parentheses represent equal-tailed 95% uncertainty intervals. Estimates represent the percentage reduction in incidence produced by removal of each risk factor.

Figure S7: Sensitivity of incidence estimates to changes in individual model parameters.*

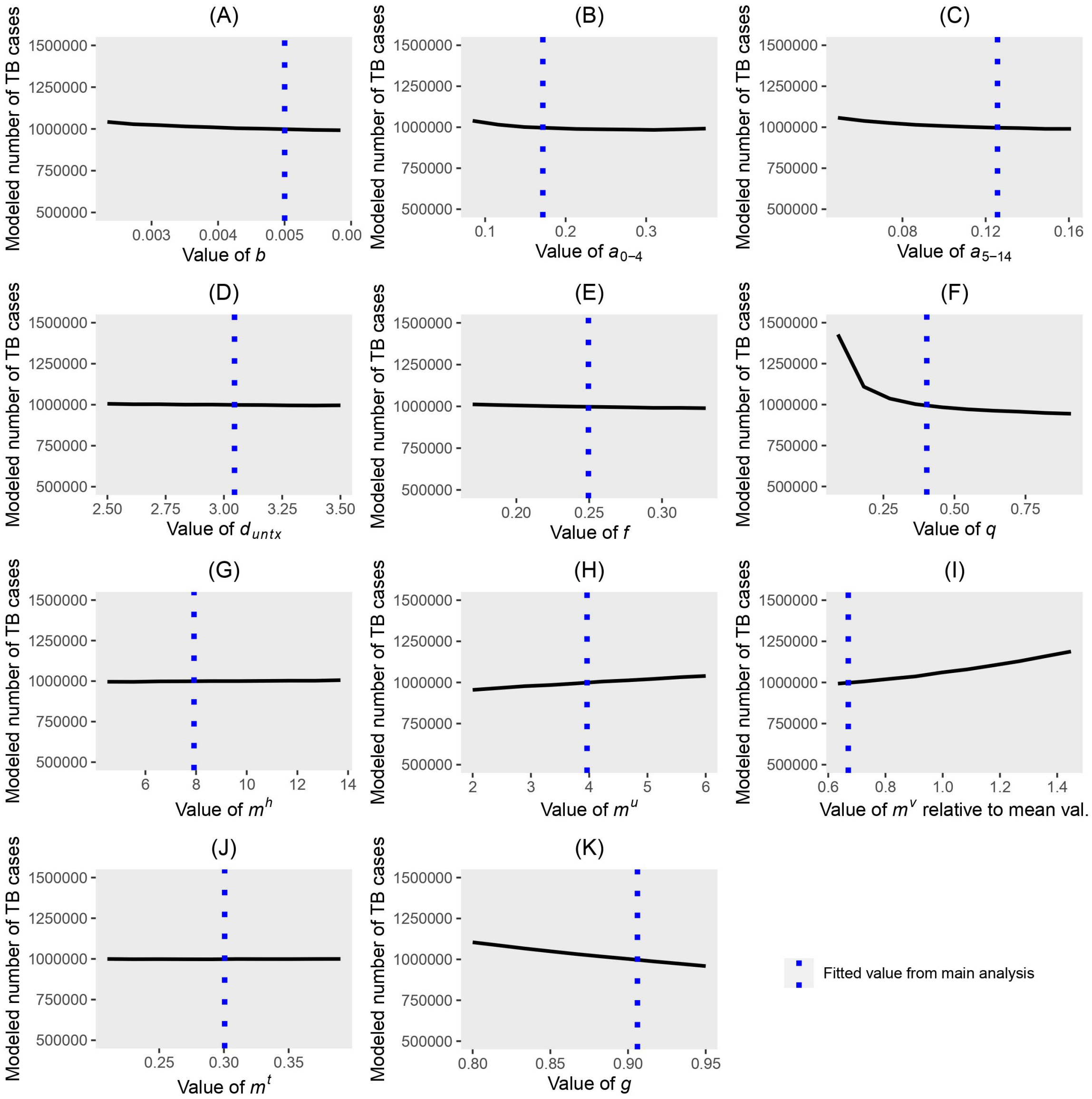

* Panel A: sensitivity analysis for b, the per-contact transmission probability for children exposed to infectious adults. Panel B: sensitivity analysis for a_{0-4}, the probability of TB disease following infectious exposure for 0-4-year-olds.Panel C: sensitivity analysis for a_{5-14}, the probability of TB disease following infectious exposure for 5-14-year-olds. Panel D: sensitivity analysis for d_{untx}, the average duration of TB disease without treatment. Panel E: sensitivity analysis for f, the fraction of total duration of disease represented by subclinical TB. Panel F: sensitivity analysis for q, the rate of contact saturation for infectious disease cases. Panel G: sensitivity analysis for m^h, the risk ratio for progression to TB disease following infectious exposure, with HIV. Panel H: sensitivity analysis for m^u, the risk ratio for TB disease following infectious exposure, with underweight. Panel I: sensitivity analysis for m_k^v, the risk ratio for TB disease following infectious exposure, with BCG vaccination. Panel J: sensitivity analysis for m^t, the risk ratio for progression to TB disease following infectious exposure, for treated HIV vs. untreated HIV. Panel K: sensitivity analysis for g, the relative probability of case detection for pediatric vs. adult cases, in countries used for model calibration.

Figure S8: Histogram of relative changes in country incidence estimates for 2019, comparing a model that assumes average contact matrices in all countries to the results of the main analysis.*

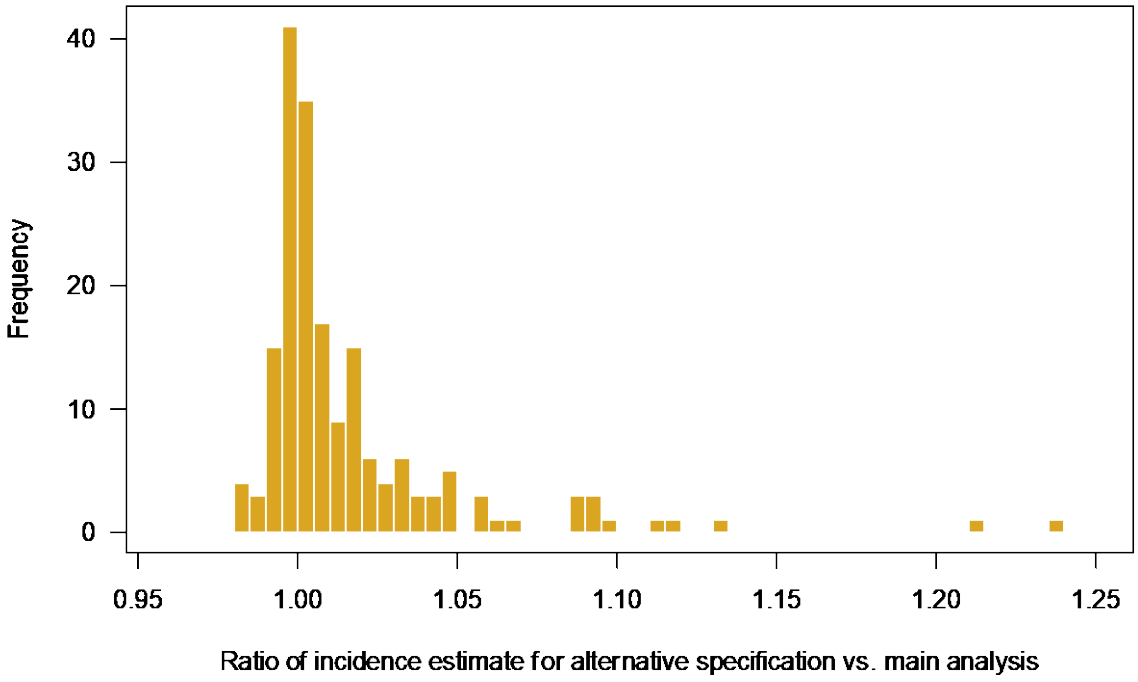

* Results calculated as incidence estimate from a model assuming common mixing patterns in all countries, divided by incidence estimate from the main analysis. Countries with a difference of >10% include United Arab Emirates (ratio = 1.24), South Korea (1.21), Greece (1.13), Puerto Rico (1.12) and Singapore (1.11).

Table S5a: Country-specific model inputs for 2019, 0-4-year-old age group.

Country	Population	CDR (overall)	Total contacts/yr	HIV prevalence	PEM prevalence	BCG coverage	ART coverage
Afghanistan	5,638,782	0.73	4,331	0.0000340	0.079	0.78	0.12
Albania	169,053	0.87	1,836	0.0000041	0.034	0.99	0.43
Algeria	5,024,616	0.8	3,488	0.0000346	0.074	0.99	1.00
Angola	5,670,471	0.66	4,134	0.0010937	0.087	0.69	0.16
Antigua and Barbuda	7,380	0.87	2,371	0.0002750	0.035	0.00	0.53
Argentina	3,741,882	0.87	2,559	0.0006268	0.019	0.93	0.67
Armenia	208,380	0.8	2,170	0.0000620	0.027	0.99	0.62
Australia	1,654,857	0.87	2,747	0.0000093	0.014	0.00	1.00
Austria	442,324	0.83	1,679	0.0000286	0.015	0.00	0.53
Azerbaijan	848,367	0.8	2,498	0.0000224	0.052	0.96	0.76
Bahamas	26,661	0.87	2,135	0.0001801	0.034	0.00	0.53
Bahrain	108,250	0.87	2,066	0.0000120	0.052	0.00	0.53
Bangladesh	14,418,746	0.81	2,791	0.0000139	0.136	0.99	0.53
Barbados	15,123	0.87	1,672	0.0000531	0.037	0.00	0.52
Belarus	556,401	0.8	1,955	0.0000802	0.036	0.97	0.88
Belgium	635,948	0.87	1,746	0.0000374	0.015	0.00	0.53
Belize	39,323	0.87	2,924	0.0005913	0.024	0.95	0.53
Benin	1,874,203	0.65	4,064	0.0006640	0.094	0.89	0.41
Bhutan	63,560	0.8	2,488	0.0001536	0.045	0.92	0.53
Bolivia	1,187,090	0.61	2,828	0.0002848	0.014	0.80	0.59
Bosnia and Herzegovina	137,597	0.65	1,313	0.0000020	0.028	0.95	0.53
Botswana	272,101	0.53	3,399	0.0014677	0.063	0.98	0.82
Brazil	14,571,950	0.87	2,108	0.0002085	0.027	0.79	0.69
Brunei Darussalam	32,650	0.87	2,287	0.0000464	0.050	0.99	0.53
Bulgaria	314,543	0.9	1,424	0.0000100	0.043	0.96	0.50
Burkina Faso	3,408,261	0.51	4,480	0.0009396	0.135	0.98	0.29
Burundi	2,018,697	0.55	4,652	0.0010112	0.082	0.93	0.39
Cote d'Ivoire	4,050,287	0.6	4,750	0.0020791	0.085	0.93	0.36
Cabo Verde	52,579	0.8	2,807	0.0009061	0.038	0.98	0.94
Cambodia	1,780,446	0.63	3,097	0.0002783	0.116	0.98	0.88
Cameroon	4,058,540	0.53	4,072	0.0018878	0.058	0.80	0.33
Canada	1,979,857	0.87	1,752	0.0000037	0.007	0.00	0.53
Central African Republic	730,857	0.47	4,174	0.0020156	0.083	0.74	0.46
Chad	2,871,727	0.6	4,319	0.0017044	0.140	0.75	0.22
Chile	1,182,968	0.87	1,962	0.0000439	0.003	0.98	0.68
China	85,041,034	0.87	1,845	0.0000465	0.029	0.99	0.53
Colombia	3,730,221	0.8	2,402	0.0000088	0.015	0.89	0.45

Country	Population	CDR (overall)	Total contacts/yr	HIV prevalence	PEM prevalence	BCG coverage	ART coverage
Comoros	122,492	0.5	3,909	0.0000118	0.098	0.94	0.60
Congo	812,951	0.59	3,720	0.0052520	0.049	0.80	0.18
Costa Rica	351,168	0.8	2,183	0.0000265	0.013	0.88	0.43
Croatia	186,911	0.9	1,562	0.0000125	0.035	0.98	0.53
Cuba	587,420	0.87	1,660	0.0001287	0.013	0.99	0.34
Cyprus	65,348	1	1,752	0.0000010	0.015	0.00	0.53
Czechia	555,583	0.87	1,719	0.0000037	0.032	0.00	0.53
North Korea	1,743,455	0.73	2,114	0.0000992	0.107	0.96	0.53
Democratic Republic of the Congo	15,502,762	0.64	4,238	0.0008450	0.105	0.73	0.28
Denmark	302,353	0.89	1,769	0.0000290	0.016	0.00	0.53
Djibouti	100,063	0.8	2,939	0.0006182	0.168	0.95	0.14
Dominican Republic	1,006,545	0.8	2,681	0.0002045	0.020	0.99	0.47
Ecuador	1,662,470	0.8	2,841	0.0000518	0.020	0.86	0.60
Egypt	12,803,817	0.69	3,630	0.0000033	0.055	0.96	0.48
El Salvador	577,564	0.8	2,600	0.0004938	0.017	0.78	0.24
Equatorial Guinea	195,125	0.63	3,901	0.0099322	0.068	0.85	0.32
Eritrea	490,685	0.61	3,874	0.0006233	0.128	0.97	0.37
Estonia	69,038	0.87	1,727	0.0000269	0.049	0.92	0.53
Eswatini	143,544	0.69	3,611	0.0138063	0.017	0.98	0.84
Ethiopia	16,560,834	0.71	3,583	0.0017799	0.110	0.69	0.48
Fiji	90,074	0.8	2,957	0.0000708	0.079	0.99	0.41
Finland	271,068	0.87	1,477	0.0000050	0.015	0.00	0.53
France	3,669,221	0.86	1,820	0.0000063	0.015	0.00	1.00
Gabon	316,297	0.48	4,136	0.0020183	0.037	0.92	0.23
Gambia	401,238	0.71	4,601	0.0012978	0.102	0.88	0.28
Georgia	274,062	0.73	2,050	0.0001152	0.019	0.96	0.56
Germany	3,987,442	0.95	1,643	0.0000099	0.008	0.00	0.53
Ghana	4,134,774	0.34	3,392	0.0006408	0.082	0.96	0.26
Greece	419,663	0.96	1,281	0.0000127	0.014	0.00	0.53
Grenada	9,064	0.87	2,371	0.0001183	0.034	0.00	0.53
Guam	13,561	0.87	2,747	0.0001583	0.060	0.00	0.53
Guatemala	2,055,787	0.8	3,212	0.0001468	0.022	0.86	0.58
Guinea	301,656	0.35	4,191	0.0010500	0.087	0.86	0.16
Guinea-Bissau	2,059,594	0.73	4,239	0.0009926	0.083	0.73	0.55
Guyana	74,578	0.8	2,738	0.0010254	0.089	0.99	0.34
Haiti	1,265,891	0.68	2,371	0.0015081	0.058	0.73	0.45
Honduras	1,010,874	0.8	2,975	0.0000340	0.015	0.88	0.53
Hungary	457,138	0.87	1,654	0.0000020	0.034	0.99	0.53

Country	Population	CDR (overall)	Total contacts/yr	HIV prevalence	PEM prevalence	BCG coverage	ART coverage
Iceland	20,712	0.87	1,945	0.0000009	0.015	0.00	0.53
India	116,781,822	0.82	2,877	0.0001425	0.180	0.92	0.53
Indonesia	23,939,761	0.67	2,765	0.0003142	0.130	0.90	0.53
Iran	7,582,802	0.8	2,858	0.0000900	0.062	0.99	0.64
Iraq	5,373,938	0.41	3,898	0.0000189	0.084	0.98	0.53
Ireland	321,521	0.87	2,058	0.0000038	0.015	0.00	0.84
Israel	846,797	0.87	3,135	0.0000322	0.013	0.00	0.53
Italy	2,382,545	0.74	1,962	0.0000178	0.013	0.00	0.83
Jamaica	233,254	0.8	2,372	0.0000732	0.028	0.97	0.41
Japan	4,905,530	0.87	2,747	0.0000051	0.026	0.99	0.53
Jordan	1,087,477	0.8	3,059	0.0000138	0.055	0.86	0.53
Kazakhstan	1,947,716	0.98	3,061	0.0000475	0.035	0.87	1.00
Kenya	7,009,450	0.6	3,324	0.0044816	0.071	0.95	0.63
Kiribati	14,959	0.8	2,747	0.0001517	0.107	0.89	0.53
Kuwait	296,871	0.87	2,205	0.0000025	0.024	0.96	0.53
Kyrgyzstan	772,576	0.87	3,270	0.0001863	0.018	0.96	0.99
Lao	794,315	0.61	3,230	0.0001936	0.093	0.79	0.57
Latvia	112,514	0.87	1,922	0.0000761	0.051	0.96	0.53
Lebanon	591,917	0.87	3,037	0.0000764	0.063	0.00	0.63
Lesotho	252,949	0.51	3,334	0.0235094	0.063	0.96	0.71
Liberia	730,151	0.54	3,822	0.0006293	0.067	0.84	0.21
Libya	632,962	0.56	2,714	0.0000588	0.060	0.74	0.42
Lithuania	147,196	0.87	1,758	0.0000545	0.049	0.97	0.35
Luxembourg	33,088	0.87	1,812	0.0000018	0.016	0.00	0.53
Madagascar	4,022,642	0.59	4,229	0.0004045	0.105	0.70	0.09
Malawi	2,877,113	0.62	3,715	0.0052431	0.044	0.91	0.74
Malaysia	2,620,281	0.87	2,624	0.0004341	0.109	0.99	0.92
Maldives	36,442	0.8	2,964	0.0000089	0.160	0.99	0.53
Mali	3,532,110	0.67	4,407	0.0008847	0.112	0.83	0.23
Malta	21,656	1	1,581	0.0000664	0.015	0.00	0.53
Mauritania	679,010	0.62	4,319	0.0000141	0.107	0.90	0.39
Mauritius	64,784	0.8	1,592	0.0000717	0.135	0.99	0.68
Mexico	11,040,956	0.8	2,599	0.0000589	0.027	0.76	0.53
Micronesia	12,171	0.8	2,747	0.0008120	0.069	0.77	0.53
Mongolia	378,629	0.31	3,379	0.0000064	0.025	0.99	0.32
Montenegro	36,874	0.87	1,990	0.0000433	0.039	0.93	0.49
Morocco	3,370,416	0.87	2,698	0.0001393	0.051	0.99	1.00
Mozambique	5,047,829	0.88	4,452	0.0239370	0.051	0.94	0.63
Myanmar	4,510,993	0.77	2,443	0.0002859	0.092	0.91	0.73

Country	Population	CDR (overall)	Total contacts/yr	HIV prevalence	PEM prevalence	BCG coverage	ART coverage
Namibia	334,399	0.64	3,810	0.0094923	0.092	0.94	0.97
Nepal	2,706,195	0.46	2,879	0.0001060	0.076	0.96	1.00
Netherlands	863,307	0.87	1,511	0.0000236	0.015	0.00	1.00
New Zealand	301,436	0.87	2,033	0.0000106	0.015	0.00	0.86
Nicaragua	661,063	0.8	2,925	0.0003521	0.019	0.98	0.69
Niger	4,642,083	0.59	4,698	0.0001705	0.143	0.70	0.42
Nigeria	33,409,364	0.27	4,130	0.0014015	0.102	0.67	0.36
North Macedonia	113,664	0.8	1,863	0.0000360	0.032	0.93	0.53
Norway	301,197	0.87	1,836	0.0000170	0.014	0.00	0.53
Oman	450,112	0.87	2,838	0.0000331	0.085	0.99	0.61
Pakistan	27,668,065	0.58	3,992	0.0000858	0.130	0.88	0.31
Panama	389,069	0.8	2,732	0.0001429	0.018	0.99	0.53
Papua New Guinea	1,095,605	0.79	3,440	0.0010798	0.116	0.52	0.49
Paraguay	697,951	0.87	2,899	0.0005668	0.010	0.87	0.52
Peru	2,810,075	0.82	2,649	0.0007608	0.009	0.81	0.65
Philippines	10,775,470	0.68	2,956	0.0008828	0.075	0.75	0.25
Poland	1,878,810	0.87	1,815	0.0000254	0.033	0.92	0.53
Portugal	406,281	0.87	1,307	0.0000125	0.015	0.00	0.53
Puerto Rico	94,123	0.87	944	0.0000744	0.031	0.00	0.53
Qatar	135,090	0.87	1,464	0.0000060	0.050	0.99	0.53
South Korea	1,969,502	0.94	1,226	0.0000346	0.015	0.98	0.53
Moldova	206,427	0.87	1,547	0.0000278	0.040	0.94	0.62
Romania	940,211	0.87	1,566	0.0000826	0.040	0.96	1.00
Russia	9,400,039	1	2,055	0.0005569	0.048	0.96	0.53
Rwanda	1,848,859	0.8	4,134	0.0008351	0.044	0.98	0.50
Saint Lucia	10,931	0.87	1,795	0.0000716	0.028	0.95	0.53
Saint Vincent and the Grenadines	7,822	0.87	2,114	0.0002442	0.033	0.99	0.53
Samoa	27,499	0.87	4,012	0.0002035	0.017	0.90	0.53
Sao Tome and Principe	31,556	0.57	4,135	0.0000129	0.082	0.95	0.53
Saudi Arabia	2,996,240	0.87	2,643	0.0000485	0.057	0.52	0.53
Senegal	2,584,884	0.7	5,187	0.0002440	0.090	0.99	0.38
Serbia	421,905	0.87	1,614	0.0000363	0.040	0.98	0.53
Seychelles	8,026	0.87	3,956	0.0000875	0.059	0.98	0.53
Sierra Leone	1,147,311	0.77	3,948	0.0004302	0.089	0.86	0.13
Singapore	249,963	0.87	1,437	0.0000159	0.062	0.98	0.73
Slovakia	283,881	0.87	1,776	0.0000203	0.035	0.00	0.53
Slovenia	102,882	0.87	1,629	0.0000031	0.034	0.00	0.53
Solomon Islands	101,949	0.8	4,282	0.0001723	0.059	0.77	0.53

Country	Population	CDR (overall)	Total contacts/yr	HIV prevalence	PEM prevalence	BCG coverage	ART coverage
Somalia	2,749,175	0.42	3,037	0.0006135	0.154	0.37	0.17
South Africa	5,785,611	0.58	2,901	0.0042958	0.050	0.84	0.47
South Sudan	1,697,855	0.65	4,185	0.0014436	0.148	0.52	0.12
Spain	2,016,181	0.94	1,416	0.0000154	0.021	0.00	1.00
Sri Lanka	1,677,468	0.61	2,389	0.0000119	0.170	0.99	0.51
Sudan	6,246,042	0.69	3,976	0.0002402	0.178	0.92	0.17
Suriname	52,451	0.8	2,647	0.0002636	0.058	0.00	0.36
Sweden	597,669	0.87	1,938	0.0000031	0.014	0.25	0.53
Switzerland	448,926	0.87	1,712	0.0000048	0.016	0.00	0.90
Syria	1,826,327	0.8	3,233	0.0000140	0.107	0.84	0.41
Tajikistan	1,346,840	0.74	4,132	0.0000207	0.081	0.98	0.96
Thailand	3,647,917	0.84	1,619	0.0002642	0.067	0.99	0.66
Timor-Leste	174,072	0.63	3,652	0.0012659	0.189	0.95	0.36
Togo	1,204,089	0.86	3,828	0.0016087	0.068	0.98	0.48
Tonga	12,240	0.87	3,396	0.0000702	0.060	0.99	0.53
Trinidad and Tobago	90,290	0.87	1,993	0.0001867	0.046	0.00	0.73
Tunisia	1,019,259	0.8	2,644	0.0000375	0.052	0.92	0.52
Turkey	6,657,922	0.87	2,405	0.0000135	0.033	0.96	0.53
Turkmenistan	675,611	0.8	3,215	0.0000162	0.055	0.98	0.53
Uganda	7,685,519	0.75	4,091	0.0026546	0.040	0.88	0.65
Ukraine	2,183,945	0.75	1,496	0.0002129	0.083	0.84	0.96
United Arab Emirates	501,230	0.87	1,542	0.0000321	0.089	0.94	0.53
United Kingdom	3,951,046	0.89	1,341	0.0000322	0.016	0.00	0.53
Tanzania	9,528,151	0.59	4,444	0.0020692	0.050	0.91	0.66
United States of America	19,604,096	0.87	1,907	0.0000193	0.006	0.00	0.53
Uruguay	237,813	0.87	2,184	0.0000351	0.017	0.99	0.53
Uzbekistan	3,434,636	0.73	3,099	0.0000637	0.077	0.99	0.91
Vanuatu	41,362	0.75	3,994	0.0001932	0.062	0.97	0.53
Venezuela	2,419,114	0.8	2,522	0.0000246	0.043	0.91	0.28
Viet Nam	7,890,956	0.6	2,655	0.0000095	0.095	0.96	0.85
Yemen	4,098,711	0.73	4,019	0.0000554	0.134	0.69	0.43
Zambia	2,901,503	0.61	3,971	0.0102997	0.058	0.95	0.76
Zimbabwe	2,138,020	0.72	3,593	0.0092147	0.040	0.95	0.71

Table S5b: Country-specific model inputs for 2019, 5-14-year-old age group.

Country	Population	CDR (overall)	Total contacts/yr	HIV prevalence	PEM prevalence	BCG coverage	ART coverage
Afghanistan	10,518,409	0.73	3,567	0.0000116	0.005	0.78	0.12
Albania	332,220	0.87	1,625	0.0000041	0.016	0.99	0.43
Algeria	8,128,254	0.8	2,562	0.0000381	0.007	0.99	1.00
Angola	9,167,133	0.66	3,031	0.0018311	0.002	0.69	0.16
Antigua and Barbuda	13,944	0.87	2,140	0.0001695	0.004	0.00	0.53
Argentina	7,272,142	0.87	2,226	0.0004878	0.005	0.93	0.67
Armenia	405,822	0.8	1,937	0.0000256	0.004	0.99	0.62
Australia	3,203,149	0.87	2,269	0.0000137	0.003	0.00	1.00
Austria	843,806	0.83	1,435	0.0000283	0.011	0.00	0.53
Azerbaijan	1,507,014	0.8	2,088	0.0000267	0.003	0.96	0.76
Bahamas	59,319	0.87	2,029	0.0003168	0.017	0.00	0.53
Bahrain	198,755	0.87	1,704	0.0000331	0.006	0.00	0.53
Bangladesh	29,953,235	0.81	2,433	0.0000106	0.006	0.99	0.53
Barbados	33,814	0.87	1,619	0.0000650	0.005	0.00	0.52
Belarus	1,053,948	0.8	1,702	0.0000749	0.005	0.97	0.88
Belgium	1,332,470	0.87	1,881	0.0000299	0.007	0.00	0.53
Belize	76,628	0.87	2,499	0.0007721	0.003	0.95	0.53
Benin	3,106,535	0.65	3,094	0.0017602	0.003	0.89	0.41
Bhutan	129,808	0.8	2,197	0.0001185	0.026	0.92	0.53
Bolivia	2,338,828	0.61	2,461	0.0001902	0.003	0.80	0.59
Bosnia and Herzegovina	347,443	0.65	1,499	0.0000017	0.007	0.95	0.53
Botswana	505,727	0.53	2,840	0.0062070	0.003	0.98	0.82
Brazil	29,767,819	0.87	1,895	0.0002532	0.003	0.79	0.69
Brunei Darussalam	65,342	0.87	2,131	0.0000418	0.004	0.99	0.53
Bulgaria	713,444	0.9	1,439	0.0000154	0.006	0.96	0.50
Burkina Faso	5,670,891	0.51	3,327	0.0013402	0.003	0.98	0.29
Burundi	3,216,260	0.55	3,347	0.0023166	0.003	0.93	0.39
Cote d'Ivoire	6,677,292	0.6	3,506	0.0047760	0.003	0.93	0.36
Cabo Verde	103,639	0.8	2,474	0.0011026	0.003	0.98	0.94
Cambodia	3,346,739	0.63	2,569	0.0006299	0.004	0.98	0.88
Cameroon	6,903,244	0.53	3,147	0.0038886	0.003	0.80	0.33
Canada	3,948,235	0.87	1,591	0.0000092	0.005	0.00	0.53
Central African Republic	1,353,228	0.47	3,401	0.0044756	0.002	0.74	0.46
Chad	4,594,750	0.6	3,150	0.0017478	0.003	0.75	0.22
Chile	2,511,592	0.87	1,885	0.0000486	0.002	0.98	0.68
China	170,236,944	0.87	1,666	0.0000435	0.014	0.99	0.53

Country	Population	CDR (overall)	Total contacts/yr	HIV prevalence	PEM prevalence	BCG coverage	ART coverage
Colombia	7,657,030	0.8	2,102	0.0000201	0.004	0.89	0.45
Comoros	211,789	0.5	3,034	0.0000058	0.002	0.94	0.60
Congo	1,421,962	0.59	2,949	0.0053493	0.002	0.80	0.18
Costa Rica	712,788	0.8	1,971	0.0000447	0.008	0.88	0.43
Croatia	414,549	0.9	1,555	0.0000137	0.005	0.98	0.53
Cuba	1,230,735	0.87	1,578	0.0000785	0.006	0.99	0.34
Cyprus	134,899	1	1,670	0.0000006	0.007	0.00	0.53
Czechia	1,125,074	0.87	1,556	0.0000028	0.005	0.00	0.53
North Korea	3,388,057	0.73	1,785	0.0000699	0.012	0.96	0.53
Democratic Republic of the Congo	24,422,116	0.64	3,083	0.0011182	0.003	0.73	0.28
Denmark	644,292	0.89	1,663	0.0000222	0.008	0.00	0.53
Djibouti	184,264	0.8	2,399	0.0027683	0.003	0.95	0.14
Dominican Republic	1,969,081	0.8	2,308	0.0004239	0.002	0.99	0.47
Ecuador	3,151,243	0.8	2,374	0.0001312	0.002	0.86	0.60
Egypt	21,162,318	0.69	2,785	0.0000051	0.007	0.96	0.48
El Salvador	1,155,733	0.8	2,277	0.0004740	0.002	0.78	0.24
Equatorial Guinea	305,980	0.63	2,722	0.0095049	0.001	0.85	0.32
Eritrea	963,588	0.61	3,318	0.0010403	0.003	0.97	0.37
Estonia	149,207	0.87	1,670	0.0000308	0.005	0.92	0.53
Eswatini	290,823	0.69	3,231	0.0297938	0.003	0.98	0.84
Ethiopia	28,647,763	0.71	2,808	0.0026742	0.004	0.69	0.48
Fiji	170,627	0.8	2,490	0.0001017	0.004	0.99	0.41
Finland	614,982	0.87	1,508	0.0000056	0.011	0.00	0.53
France	7,925,163	0.86	1,797	0.0000076	0.007	0.00	1.00
Gabon	491,458	0.48	2,893	0.0029928	0.002	0.92	0.23
Gambia	634,735	0.71	3,253	0.0018768	0.004	0.88	0.28
Georgia	526,549	0.73	1,848	0.0000394	0.003	0.96	0.56
Germany	7,537,542	0.95	1,113	0.0000194	0.007	0.00	0.53
Ghana	7,230,820	0.34	2,733	0.0022734	0.003	0.96	0.26
Greece	1,035,780	0.96	1,472	0.0000077	0.006	0.00	0.53
Grenada	17,498	0.87	2,140	0.0000922	0.004	0.00	0.53
Guam	26,813	0.87	2,269	0.0001462	0.005	0.00	0.53
Guatemala	3,897,181	0.8	2,731	0.0001271	0.002	0.86	0.58
Guinea	508,081	0.35	3,154	0.0038381	0.002	0.86	0.16
Guinea-Bissau	3,489,475	0.73	3,219	0.0015130	0.002	0.73	0.55
Guyana	144,049	0.8	2,357	0.0015797	0.003	0.99	0.34
Haiti	2,434,985	0.68	2,140	0.0030308	0.003	0.73	0.45
Honduras	2,025,958	0.8	2,593	0.0000552	0.002	0.88	0.53

Country	Population	CDR (overall)	Total contacts/yr	HIV prevalence	PEM prevalence	BCG coverage	ART coverage
Hungary	940,669	0.87	1,472	0.0000037	0.004	0.99	0.53
Iceland	45,899	0.87	1,992	0.0000013	0.008	0.00	0.53
India	246,934,918	0.82	2,500	0.0001880	0.025	0.92	0.53
Indonesia	47,005,067	0.67	2,361	0.0001436	0.013	0.90	0.53
Iran	12,859,391	0.8	2,153	0.0000278	0.009	0.99	0.64
Iraq	9,572,005	0.41	3,122	0.0000108	0.009	0.98	0.53
Ireland	711,145	0.87	2,118	0.0000025	0.010	0.00	0.84
Israel	1,528,878	0.87	2,603	0.0000286	0.008	0.00	0.53
Italy	5,589,844	0.74	1,873	0.0000194	0.013	0.00	0.83
Jamaica	460,511	0.8	2,075	0.0001040	0.004	0.97	0.41
Japan	11,044,517	0.87	2,269	0.0000071	0.006	0.99	0.53
Jordan	2,302,873	0.8	2,915	0.0000077	0.008	0.86	0.53
Kazakhstan	3,410,153	0.98	2,517	0.0000404	0.003	0.87	1.00
Kenya	13,596,067	0.6	2,838	0.0075782	0.005	0.95	0.63
Kiribati	27,191	0.8	2,269	0.0001156	0.004	0.89	0.53
Kuwait	610,151	0.87	2,099	0.0000033	0.012	0.96	0.53
Kyrgyzstan	1,312,687	0.87	2,596	0.0001780	0.004	0.96	0.99
Lao	1,521,640	0.61	2,720	0.0002174	0.005	0.79	0.57
Latvia	198,431	0.87	1,489	0.0000546	0.005	0.96	0.53
Lebanon	1,161,203	0.87	2,484	0.0000399	0.007	0.00	0.63
Lesotho	437,062	0.51	2,554	0.0290115	0.003	0.96	0.71
Liberia	1,281,972	0.54	2,967	0.0013891	0.002	0.84	0.21
Libya	1,269,348	0.56	2,442	0.0000332	0.008	0.74	0.42
Lithuania	270,509	0.87	1,518	0.0000346	0.005	0.97	0.35
Luxembourg	63,626	0.87	1,825	0.0000013	0.008	0.00	0.53
Madagascar	6,864,013	0.59	3,173	0.0002497	0.004	0.70	0.09
Malawi	5,220,559	0.62	2,961	0.0115364	0.004	0.91	0.74
Malaysia	4,950,212	0.87	2,169	0.0003861	0.011	0.99	0.92
Maldives	69,002	0.8	2,262	0.0000113	0.030	0.99	0.53
Mali	5,767,053	0.67	3,276	0.0011846	0.005	0.83	0.23
Malta	41,443	1	1,380	0.0000611	0.010	0.00	0.53
Mauritania	1,126,241	0.62	3,208	0.0000211	0.004	0.90	0.39
Mauritius	154,361	0.8	1,636	0.0000458	0.008	0.99	0.68
Mexico	22,364,802	0.8	2,334	0.0000911	0.008	0.76	0.53
Micronesia	23,628	0.8	2,269	0.0015597	0.003	0.77	0.53
Mongolia	614,667	0.31	2,554	0.0000044	0.003	0.99	0.32
Montenegro	77,108	0.87	1,822	0.0000361	0.008	0.93	0.49
Morocco	6,465,838	0.87	2,343	0.0001359	0.008	0.99	1.00
Mozambique	8,424,965	0.88	3,292	0.0211497	0.002	0.94	0.63

Country	Population	CDR (overall)	Total contacts/yr	HIV prevalence	PEM prevalence	BCG coverage	ART coverage
Myanmar	9,494,520	0.77	2,206	0.0009697	0.005	0.91	0.73
Namibia	586,226	0.64	3,021	0.0161306	0.004	0.94	0.97
Nepal	5,749,784	0.46	2,490	0.0001899	0.010	0.96	1.00
Netherlands	1,852,308	0.87	1,584	0.0000217	0.008	0.00	1.00
New Zealand	634,448	0.87	1,925	0.0000115	0.005	0.00	0.86
Nicaragua	1,293,090	0.8	2,522	0.0002352	0.003	0.98	0.69
Niger	6,976,482	0.59	3,251	0.0003929	0.003	0.70	0.42
Nigeria	54,386,669	0.27	3,088	0.0011294	0.007	0.67	0.36
North Macedonia	227,891	0.8	1,666	0.0000178	0.009	0.93	0.53
Norway	635,424	0.87	1,755	0.0000139	0.012	0.00	0.53
Oman	664,137	0.87	1,923	0.0000392	0.011	0.99	0.61
Pakistan	48,247,549	0.58	3,097	0.0000268	0.010	0.88	0.31
Panama	748,093	0.8	2,332	0.0001192	0.004	0.99	0.53
Papua New Guinea	2,018,684	0.79	2,792	0.0014053	0.004	0.52	0.49
Paraguay	1,357,631	0.87	2,479	0.0004191	0.003	0.87	0.52
Peru	5,400,259	0.82	2,213	0.0004156	0.002	0.81	0.65
Philippines	22,174,027	0.68	2,586	0.0001955	0.007	0.75	0.25
Poland	3,874,605	0.87	1,831	0.0000249	0.009	0.92	0.53
Portugal	948,846	0.87	1,383	0.0000130	0.010	0.00	0.53
Puerto Rico	382,842	0.87	1,806	0.0000523	0.005	0.00	0.53
Qatar	249,829	0.87	1,257	0.0000086	0.009	0.99	0.53
South Korea	4,559,906	0.94	1,310	0.0000280	0.004	0.98	0.53
Moldova	436,035	0.87	1,478	0.0000137	0.006	0.94	0.62
Romania	2,075,619	0.87	1,504	0.0001279	0.007	0.96	1.00
Russia	17,082,689	1	1,725	0.0005423	0.007	0.96	0.53
Rwanda	3,172,380	0.8	3,116	0.0018224	0.002	0.98	0.50
Saint Lucia	22,356	0.87	1,623	0.0000601	0.004	0.95	0.53
Saint Vincent and the Grenadines	16,721	0.87	2,006	0.0001782	0.004	0.99	0.53
Samoa	47,130	0.87	3,074	0.0002212	0.004	0.90	0.53
Sao Tome and Principe	59,079	0.57	3,436	0.0000121	0.003	0.95	0.53
Saudi Arabia	5,523,027	0.87	2,233	0.0000420	0.006	0.52	0.53
Senegal	4,396,425	0.7	4,039	0.0006566	0.003	0.99	0.38
Serbia	941,380	0.87	1,574	0.0000393	0.007	0.98	0.53
Seychelles	15,178	0.87	3,050	0.0001381	0.005	0.98	0.53
Sierra Leone	2,034,568	0.77	3,082	0.0017070	0.003	0.86	0.13
Singapore	465,803	0.87	1,162	0.0000189	0.006	0.98	0.73
Slovakia	563,476	0.87	1,558	0.0000124	0.006	0.00	0.53
Slovenia	211,111	0.87	1,549	0.0000053	0.007	0.00	0.53

Country	Population	CDR (overall)	Total contacts/yr	HIV prevalence	PEM prevalence	BCG coverage	ART coverage
Solomon Islands	166,858	0.8	3,140	0.0002089	0.003	0.77	0.53
Somalia	4,413,165	0.42	2,484	0.0007976	0.003	0.37	0.17
South Africa	11,177,767	0.58	2,425	0.0159026	0.003	0.84	0.47
South Sudan	2,900,448	0.65	3,151	0.0018988	0.003	0.52	0.12
Spain	4,796,484	0.94	1,539	0.0000138	0.009	0.00	1.00
Sri Lanka	3,430,899	0.61	2,162	0.0000147	0.032	0.99	0.51
Sudan	10,945,919	0.69	3,077	0.0003049	0.010	0.92	0.17
Suriname	104,005	0.8	2,311	0.0002510	0.003	0.00	0.36
Sweden	1,171,291	0.87	1,758	0.0000027	0.013	0.25	0.53
Switzerland	834,683	0.87	1,452	0.0000092	0.008	0.00	0.90
Syria	3,475,306	0.8	2,563	0.0000054	0.010	0.84	0.41
Tajikistan	2,108,567	0.74	2,965	0.0000114	0.004	0.98	0.96
Thailand	8,065,535	0.84	1,584	0.0001992	0.005	0.99	0.66
Timor-Leste	308,157	0.63	2,890	0.0009042	0.007	0.95	0.36
Togo	2,109,550	0.86	3,031	0.0031272	0.003	0.98	0.48
Tonga	24,410	0.87	3,014	0.0000776	0.004	0.99	0.53
Trinidad and Tobago	192,346	0.87	1,893	0.0002489	0.004	0.00	0.73
Tunisia	1,813,896	0.8	2,176	0.0000219	0.008	0.92	0.52
Turkey	13,607,970	0.87	2,216	0.0000068	0.004	0.96	0.53
Turkmenistan	1,152,074	0.8	2,576	0.0000239	0.004	0.98	0.53
Uganda	12,899,823	0.75	3,097	0.0070058	0.002	0.88	0.65
Ukraine	4,828,527	0.75	1,534	0.0003104	0.007	0.84	0.96
United Arab Emirates	937,658	0.87	1,382	0.0000205	0.009	0.94	0.53
United Kingdom	7,998,863	0.89	1,339	0.0000195	0.018	0.00	0.53
Tanzania	15,903,650	0.59	3,285	0.0047035	0.002	0.91	0.66
United States of America	41,435,218	0.87	1,803	0.0000172	0.006	0.00	0.53
Uruguay	470,389	0.87	1,921	0.0000373	0.004	0.99	0.53
Uzbekistan	6,063,388	0.73	2,479	0.0000418	0.003	0.99	0.91
Vanuatu	74,724	0.75	3,159	0.0002196	0.003	0.97	0.53
Venezuela	5,395,213	0.8	2,526	0.0000547	0.006	0.91	0.28
Viet Nam	14,501,606	0.6	2,185	0.0000427	0.014	0.96	0.85
Yemen	7,339,584	0.73	3,220	0.0000318	0.011	0.69	0.43
Zambia	5,039,967	0.61	3,108	0.0118351	0.002	0.95	0.76
Zimbabwe	4,036,215	0.72	3,007	0.0199349	0.004	0.95	0.71

Supplement citations

1. Sun SJ, Bennett DE, Flood J, Loeffler AM, Kammerer S, Ellis BA. Identifying the sources of tuberculosis in young children: a multistate investigation. Emerging infectious diseases. 2002;8(11):1216.

2. Frascella B, Richards AS, Sossen B, Emery JC, Odone A, Law I, et al. Subclinical tuberculosis disease-a review and analysis of prevalence surveys to inform definitions, burden, associations and screening methodology. Clin Infect Dis. 2020.

3. Dowdy DW, Basu S, Andrews JR. Is Passive Diagnosis Enough? The Impact of Subclinical Disease on Diagnostic Strategies for Tuberculosis. Am J Resp Crit Care. 2013;187(5):543-51.

4. Gelman A. Prior distributions for variance parameters in hierarchical models. Bayesian Analysis. 2006;1(3):515-33.

5. Carpenter B, Gelman A, Hoffman MD, Lee D, Goodrich B, Betancourt M, et al. Stan: A probabilistic programming language. Journal of Statistical Software. 2017;76(1).

6. Stan Development Team. RStan: the R interface to Stan. R package version 2.14.1 (http://mc-stan.org). 2016.

7. Hoffman MD, Gelman A. The No-U-Turn sampler: adaptively setting path lengths in Hamiltonian Monte Carlo. J Mach Learn Res. 2012;15(1):1593-623.

8. United Nations Population Division. World Population Prospects 2019, Online Edition, Rev 1 [https://population.un.org/wpp/Download/Standard/Population/, accessed Dec 25 2020]. Geneva: United Nations, Department of Economic and Social Affairs; 2019.

9. WHO Global TB Database [retrieved from http://www.who.int/tb/country/data/download/en/, Dec 25 2020] [Internet]. WHO Global TB Programme. 2020.

10. Prem K, van Zandvoort K, Klepac P, Eggo RM, Davies NG, Cook AR, et al. Projecting contact matrices in 177 geographical regions: an update and comparison with empirical data for the COVID-19 era. medRxiv. 2020.

11. Global Burden of Disease Collaborative Network. Global Burden of Disease Study 2019 (GBD 2019) Results [http://ghdx.healthdata.org/gbd-results-tool, accessed

Dec 25 2020]. Seattle, United States: Institute for Health Metrics and Evaluation; 2020.

12. UNAIDS. AIDSinfo Epidemiological Estimates Database [retrieved from https://aidsinfo.unaids.org/, Feb 6 2021]. Geneva, Switzerland: UNAIDS; 2021.
13. World Health Organisation. WHO-UNICEF estimates of BCG coverage. WHO vaccine-preventable diseases: monitoring system 2020 global summary [https://apps.who.int/immunization_monitoring/globalsummary/timeseries/tswucoveragebcg.html, accessed January 23, 2021]. Geneva, Switzerland; 2020.
14. Tiemersma EW, van der Werf MJ, Borgdorff MW, Williams BG, Nagelkerke NJD. Natural history of tuberculosis: duration and fatality of untreated pulmonary tuberculosis in HIV negative patients: a systematic review. PLOS ONE. 2011;6(4):e17601-e.
15. Sutherland I, Fayers PM. The association of the risk of tuberculous infection with age. Bulletin of the International Union Against Tuberculosis. 1975;50(1):70-81.
16. Styblo K, Meijer J. The quantified increase of the tuberculosis infection rate in a low prevalence country to be expected if the existing MMR programme were discontinued. Bulletin of the International Union of Tuberculosis. 1980;55:3-8.
17. Martinez L, Cords O, Horsburgh CR, Andrews JR, Acuna-Villaorduna C, Ahuja SD, et al. The risk of tuberculosis in children after close exposure: a systematic review and individual-participant meta-analysis. The Lancet. 2020;395(10228):973-84.
18. Dodd P, Prendergast A, Beecroft C, Kampmann B, Seddon J. The impact of HIV and antiretroviral therapy on TB risk in children: a systematic review and meta-analysis. Thorax. 2017;72(6):559-75.
19. Lonnroth K, Williams BG, Cegielski P, Dye C. A consistent log-linear relationship between tuberculosis incidence and body mass index. Int J Epidemiol. 2010;39(1):149-55.
20. Mangtani P, Abubakar I, Ariti C, Beynon R, Pimpin L, Fine PEM, et al. Protection by BCG Vaccine Against Tuberculosis: A Systematic Review of Randomized Controlled Trials. Clinical Infectious Diseases. 2013;58(4):470-80.
